# Elongation/Termination Factor Exchange Mediated by PP1 Phosphatase Orchestrates Transcription Termination

**DOI:** 10.1016/j.celrep.2018.09.007

**Published:** 2018-10-02

**Authors:** Tea Kecman, Krzysztof Kuś, Dong-Hyuk Heo, Katie Duckett, Adrien Birot, Sabrina Liberatori, Shabaz Mohammed, Lucia Geis-Asteggiante, Carol V. Robinson, Lidia Vasiljeva

**Affiliations:** 1 Department of Biochemistry, University of Oxford, Oxford OX1 3QU, UK; 2 Department of Chemistry, University of Oxford, Oxford OX1 3QU, UK

**Keywords:** RNA polymerase II, C-terminal domain, CTD, PP1 phosphatase, transcription termination, CTD phosphorylation, Spt5, CTD interacting domain, CID

## Abstract

Termination of RNA polymerase II (Pol II) transcription is a key step that is important for 3′ end formation of functional mRNA, mRNA release, and Pol II recycling. Even so, the underlying termination mechanism is not yet understood. Here, we demonstrate that the conserved and essential termination factor Seb1 is found on Pol II near the end of the RNA exit channel and the Rpb4/7 stalk. Furthermore, the Seb1 interaction surface with Pol II largely overlaps with that of the elongation factor Spt5. Notably, Seb1 co-transcriptional recruitment is dependent on Spt5 dephosphorylation by the conserved PP1 phosphatase Dis2, which also dephosphorylates threonine 4 within the Pol II heptad repeated C-terminal domain. We propose that Dis2 orchestrates the transition from elongation to termination phase during the transcription cycle by mediating elongation to termination factor exchange and dephosphorylation of Pol II C-terminal domain.

## Highlights

• Phosphorylated Tyr1 and Thr4 of the Pol II CTD are enriched at gene 3′ ends
• PP1 phosphatase Dis2 specifically dephosphorylates Thr4
• Dis2 regulates recruitment of termination factor Seb1 by dephosphorylating Spt5
• Dis2 orchestrates Pol II release and factor exchange at the end of transcription

## Graphical Abstract

**Figure:**
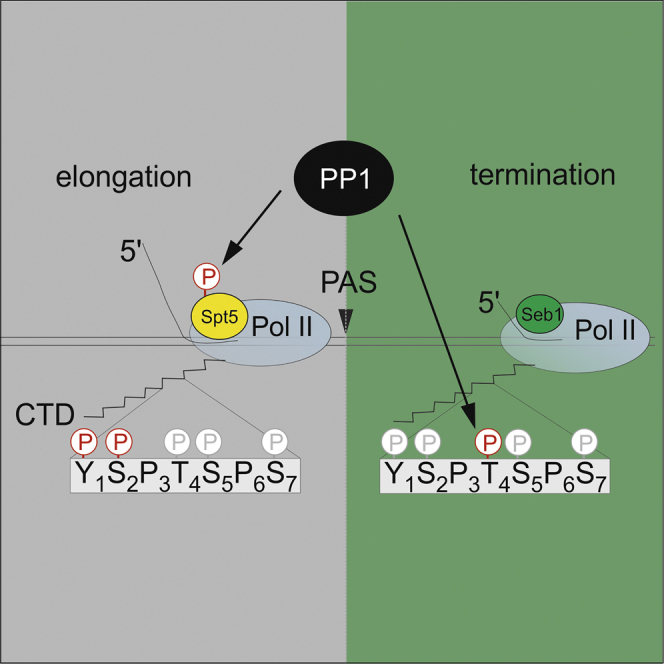

## Teaser

Timely and efficient transcription termination is essential for release of functional mRNAs as well as for Pol II recycling. Kecman et al. demonstrate that the conserved PP1 phosphatase Dis2 regulates transcription termination in fission yeast by mediating elongation to termination factor exchange and by dephosphorylating Pol II C-terminal domain.

## Introduction

Transcription of mRNAs is a tightly regulated process that requires the recruitment of RNA polymerase II (Pol II) to promoters, transcription initiation, elongation, and termination. An exchange between elongation and polyadenylation/termination factors regulates the elongation-to-termination transition, once Pol II transcribes through the polyadenylation site (PAS) at the 3′ end of genes. This process is thought to include: (1) dissociation of the elongation factors Spt4/5, a complex also known as DSIF (DRB Sensitivity Inducing Factor) (Baejen et al., 2017), (2) recruitment of the multi-subunit cleavage and polyadenylation factor (CPF) and the cleavage factor IA to PAS that is essential for pre-mRNA cleavage and polyadenylation (Birse et al., 1998, Connelly and Manley, 1988, Gross and Moore, 2001a, Gross and Moore, 2001b, Minvielle-Sebastia et al., 1997, Zaret and Sherman, 1982), and (3) association of the termination factors to dislodge Pol II from the DNA template (Kim et al., 2004, Zhang and Gilmour, 2006).

Although timely and efficient transcription termination by Pol II is essential for the production and release of functional mRNAs as well as for Pol II recycling, it remains poorly understood. To shed light on this important problem, it is key to understand how elongation factors are exchanged for termination factors and how termination factors modulate the release of Pol II from DNA.

Several highly conserved key termination factors have been identified. A characteristic feature of these factors is that they directly interact with the phosphorylated C-terminal domain (CTD) of the largest subunit of Pol II (Rpb1) via their conserved CTD-interacting domain (CID). The CTD consists of heptad repeats (Y^1^S^2^P^3^T^4^S^5^P^6^S^7^) where Tyr1, Ser2, Ser5, Ser7, and Thr4 can be reversibly phosphorylated during the transcription cycle (Buratowski, 2003, Eick and Geyer, 2013, Kobor and Greenblatt, 2002). CID-containing termination factors interact with the CTD in a phosphorylation-dependent manner. In the fission yeast *Schizosaccharomyces pombe* (*S. pombe*), there are two essential CID-containing factors, Pcf11 and Seb1, and one non-essential factor Rhn1 (homologous to Rtt103 in *Saccharomyces cerevisiae* [*S. cerevisiae*]). All three termination factors interact with Ser2P CTD (Kim et al., 2004, Lemay et al., 2016, Meinhart and Cramer, 2004, Noble et al., 2005, Wittmann et al., 2017). Termination also relies on recognition of the PAS at the 3′ end of nascent transcripts by the CPF complex. The CPF complex is composed of three functionally distinct modules: the polyadenylation, nuclease, and phosphatase modules. The polyadenylation module is involved in the recognition of the PAS (Casañal et al., 2017, Clerici et al., 2017, Schönemann et al., 2014, Sun et al., 2018) and in the polyadenylation of the mRNA 3′ end following cleavage by the nuclease module. The phosphatase module is known to contain two phosphatases, Ssu72 and a PP1 phosphatase (Glc7 in *S. cerevisiae* and Dis2 in *S. pombe*) (Krishnamurthy et al., 2004, Nedea et al., 2003, Vanoosthuyse et al., 2014). Although transcription termination was reported to be affected in Glc7 mutant (He and Moore, 2005, Schreieck et al., 2014), the molecular basis behind this is not well understood. In mammals silencing of PP1-cofactor PNUTS causes readthrough transcription at the 3′ ends. However, whether PP1 or its catalytic activity are involved and how is not clear (Austenaa et al., 2015).

In this study, we demonstrate that the PP1 phosphatase Dis2 is important for transcription termination of Pol II genome-wide in fission yeast. We show that loss of Dis2 leads to a global increase in levels of Thr4P at the end of the transcription cycle, but other CTD modifications are unaffected either *in vitro* or *in vivo*. Surprisingly, we show that Seb1 recruitment to chromatin is compromised in *dis2Δ*, suggesting that the termination defect observed in this mutant might be due to insufficient levels of Seb1. To understand the molecular basis of transcription termination, we investigated how Seb1 interacts with Pol II using cross-linking coupled to mass spectrometry. Unexpectedly, Seb1 is found close to multiple subunits of Pol II, showing that Seb1 interactions with Pol II are not solely restricted to the Rpb1 CTD. Interestingly, Seb1 contacts Pol II in close proximity to the region known to interact with the elongation factors Spt4/5, suggesting that Seb1 and Spt5 compete for interaction with the polymerase. Spt5, like Rpb1, has a C-terminal region (CTR) that is composed of 18 tandem repeats of the consensus T^1^P^2^A^3^W^4^N^5^S^6^G^7^S^8^K^9^, where Thr1 is phosphorylated by the Pol II CTD kinase Cdk9 early during the transcription cycle (Booth et al., 2018, Pei and Shuman, 2003, Swanson et al., 1991, Viladevall et al., 2009). A recent study has shown that Spt5 is dephosphorylated before the PAS by Dis2 and has proposed that this dephosphorylation facilitates termination, although it remains unclear how (Parua et al., 2018). Here, we have investigated the relationship between Spt5 and Seb1 and demonstrated that Seb1 recruitment is dependent on dephosphorylation of Spt5 by Dis2. We have further revealed that Dis2 orchestrates the transition from elongation to termination and Pol II release by de-phosphorylating both Thr4P of the Rpb1 CTD as well as Spt5. This, in turn, allows the efficient recruitment of Seb1.

## Results

### Tyr1 and Thr4 CTD Phospho-Marks Are Enriched at Gene 3′ Ends

**Figure 1:**
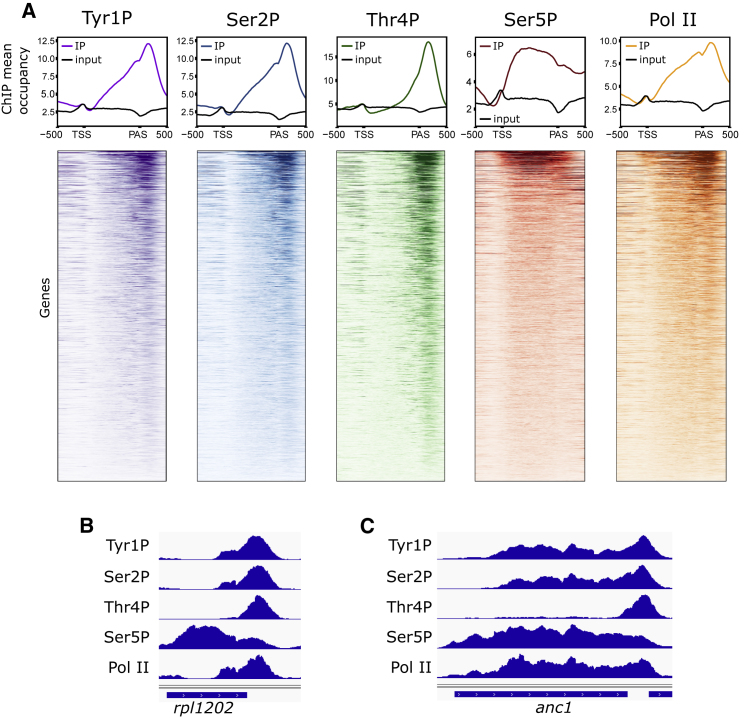
Differential Distribution of Phospho-Marks on Protein-Coding Genes (A) Averaged occupancy profiles of Pol II (8WG16), phospho-Pol II, and input from ChIP-seq on all 4,105 yeast protein coding-genes (upper panel). Color-coded heatmaps illustrating the phospho-Pol II profiles across single genes (lower panel). Profiles are aligned to the TSS (transcription start site) and PAS (polyadenylation site) as indicated. (B and C) ChIP-seq profiles across *rpl1202* (B) and *anc1* (C) genes.

To understand how different Pol II phospho-marks are distributed genome-wide during transcription in fission yeast, we performed chromatin immunoprecipitation (ChIP) using antibodies against Tyr1P, Thr4P, Ser2P, Ser5P followed by high-throughput sequencing (Figures 1A–1C). Pol II occupancy was monitored using an antibody specific for the CTD (8WG16). Ser5P is enriched across the gene body, whereas Ser2P increases toward the 3′ end of genes consistent with the reported distribution of this phospho-mark in fission yeast (Coudreuse et al., 2010) and other organisms. Tyr1P levels are also increased toward the 3′ end, peaking after the PAS similar to Ser2P suggesting that both marks are likely to contribute to the regulation of events during elongation and 3′ end formation. Interestingly, Thr4P gives an even more 3′ end distal profile, peaking after the PAS, further downstream as compared to Ser2P and Tyr1P. This may be consistent with the possible global role for Thr4P late during transcription. Notably, the Thr4P profile we observe in fission yeast resembles the distribution in mammals (Hintermair et al., 2012, Schlackow et al., 2017) but differs from the pattern observed in *S. cerevisiae* where Thr4P is present within the gene body (Mayer et al., 2012), even though it is also present after the PAS (Harlen et al., 2016, Milligan et al., 2016).

### PP1 Phosphatase Regulates Levels of Thr4P on Pol II CTD

**Figure 2:**
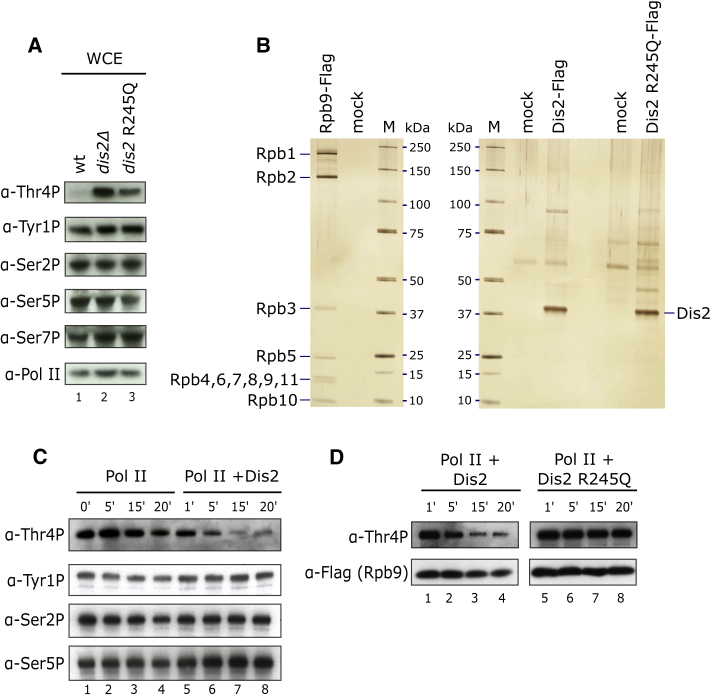
Dis2 Is a Thr4P Phosphatase (A) Deletion of *dis2* or expression of the phosphatase-defective *dis2-*R245Q allele increase the level of Thr4P. Whole-cell extracts were analyzed by western blot using antibodies specific for Thr4P, Tyr1P, Ser2P, Ser5P, Ser7P, and Pol II (8WG16). (B) Silver-stained SDS-PAGE analysis of Pol II, Dis2, and Dis2 R245Q purified from yeast. (C) Purified Pol II was incubated with Dis2, and levels of phosphorylated Pol II were assessed by western blot using antibodies specific for Thr4P, Tyr1P, Ser2P, and Ser5P. (D) Purified Pol II was incubated with Dis2 or Dis2 R245Q, and levels of Thr4P Pol II were assessed by western blot with anti-CTD and Flag antibodies. See also Figure S1.

To understand how Thr4P contributes to transcription regulation, we first wanted to identify enzymes involved in maintaining Thr4P levels. Conserved phosphatases Ssu72 and PP1 are recruited late during transcription via their interaction with CPF. In fission yeast, there are two PP1 phosphatases, Dis2 and Sds21 (Ohkura et al., 1989). To test whether they contribute to regulation of Thr4P, we analyzed the levels of phosphorylated CTD in whole-cell extract in the absence of Dis2 (Figure 2A, lines 1 and 2) or Sds21 (Figure S1A). Strikingly, we observed an increase in Thr4P in the *dis2Δ* mutant, whereas other phospho-marks were not affected. The same phenotype was observed in a strain carrying a *dis2* R245Q allele, in line with previous studies, showing that this point mutant has reduced catalytic activity (Kinoshita et al., 1990) (Figure 2A, lines 1–3). Deletion of Sds21 had no effect on phosphorylated CTD, suggesting that Dis2 is the only PP1 isoform targeting Pol II in *S. pombe*. No change in Thr4P was observed in *ssu72Δ* (data not shown). Levels of Thr4P in the wild-type (WT) and *sds21Δ* strains are barely detectable when compared to *dis2Δ* (Figures 2A and S1B); however, Thr4P can be detected in these strains using longer exposure (Figure S1A). We next used an *in vitro* approach to test whether Dis2 is directly responsible for dephosphorylation of Pol II. Since phosphatases rely on regulatory co-factors that provide specificity in targeting their substrate (Shi, 2009), we purified endogenous Dis2 in complex with associated proteins as well as the catalytically inactive variant (Dis2 R245Q) from fission yeast (Figure 2B). We then assayed phosphatase activity using native Pol II purified from yeast as a substrate (Figure 2B). In agreement with our *in vivo* data, addition of Dis2 resulted in dephosphorylation of Thr4P Pol II but not Tyr1P, Ser2P, or Ser5P (Figure 2C). In contrast, addition of the inactive enzyme did not affect Thr4P levels (Figure 2D). Taken together, these results show that Dis2 is a CTD phosphatase that specifically targets Thr4P on Pol II.

### Loss of Dis2 Leads to Defective Transcription Termination

**Figure 3:**
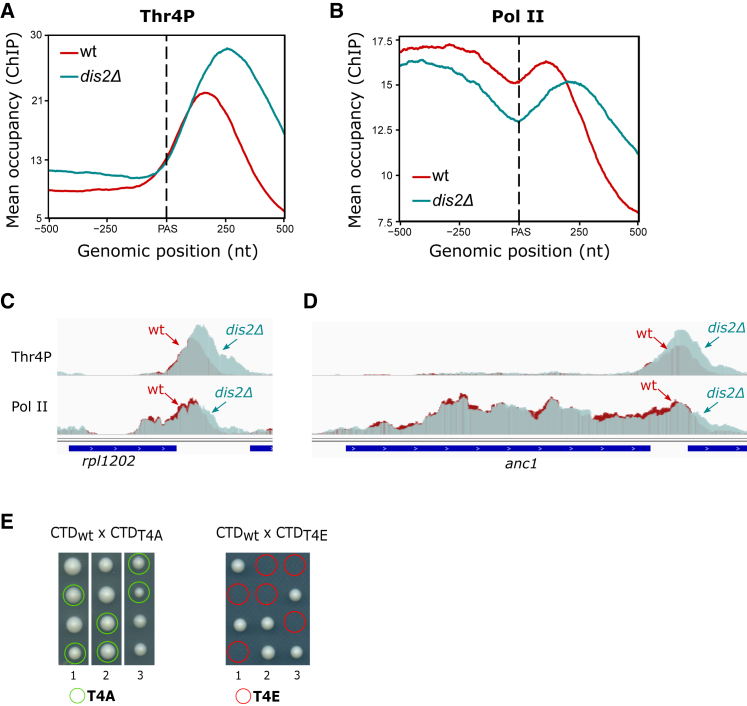
Increased Thr4P Downstream of PAS upon Loss of Dis2 Coincides with Transcription Termination Defect (A and B) Spike-in normalized ChIP-seq meta-profiles of Thr4P (A) and Pol II-8WG16 (B) around the PAS over a set of 1,735 representative non-overlapping protein-coding genes (see STAR Methods), averaged from two biological replicas. (C and D) Thr4P and Pol II profiles across *rpl1202* (C) and *anc1* (D) genes. (E) Results from a tetrad dissection of heterozygous T4A-CTD (left) and T4E-CTD (right) mutants. Three dissections are shown. See also Figure S2.

To further understand Dis2 function in Pol II transcription, we analyzed the distribution of Thr4P upon Dis2 loss using calibrated ChIP sequencing (ChIP-seq). Deletion of *dis2* caused a pronounced increase of Thr4P within the region downstream of PAS (Figures 3 and S2; p value <0.001, two-sided Wilcoxon rank-sum test) as evident from the metagene plots as well as from analysis of single genes (Figures 3A–3D, S2A, and S2B). Strikingly, increased Thr4P levels correlate with Pol II accumulation downstream of the PAS in *dis2*Δ compared to WT suggesting that Pol II is less efficiently released from DNA upon loss of Dis2 (Figures 3B–3D).

To determine the importance of Thr4 phosphorylation during the transcription cycle, we replaced Thr4 residues in all the 25 canonical repeats of the CTD either with an alanine (T4A), preventing phosphorylation at this position *in vivo*, or with a glutamic acid (T4E), mimicking a constitutively phosphorylated Thr4 (Figure 3E). The T4A mutant is viable in agreement with previous work (Schwer and Shuman, 2011), where a truncated CTD with 14 canonical repeats carrying the T4A mutation was also viable. The T4E mutant is lethal, suggesting that tight control of where and when Thr4 phospho-mark is placed and kept is important for cell survival.

### Recruitment of Termination Factors Is Impaired upon Dis2 Deletion

**Figure 4:**
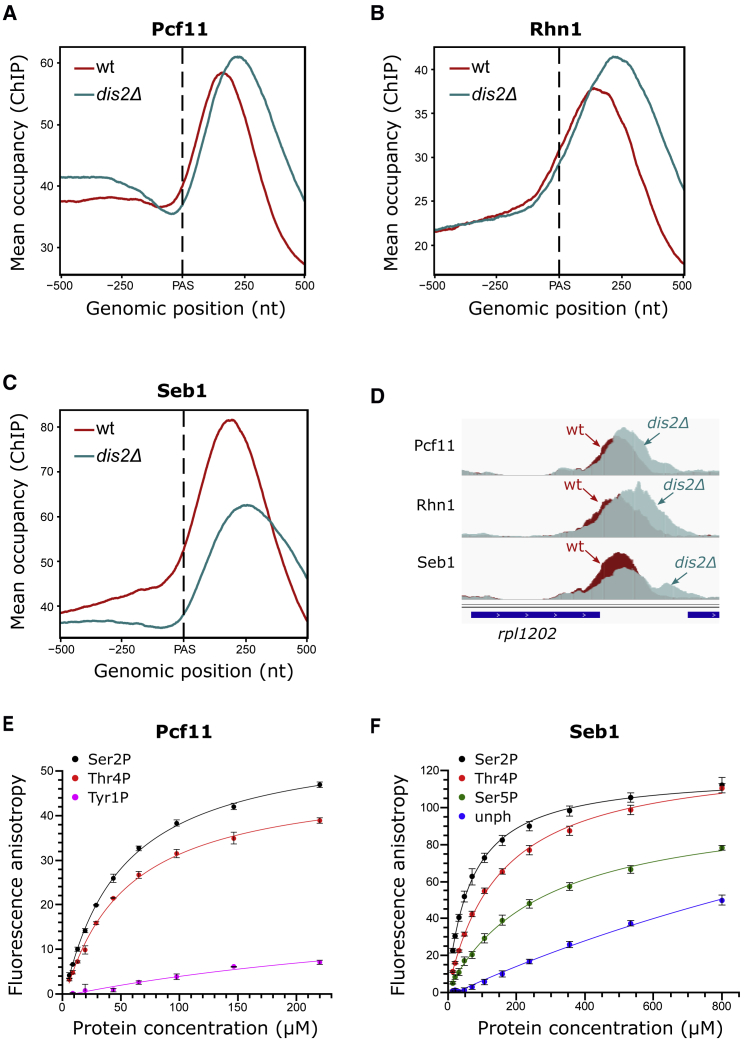
Lack of Dis2 Activity Leads to Global Decrease of the Termination Factor Seb1 (A–C) Spike-in normalized ChIP-seq meta-profiles of Pcf11 (A), Rhn1 (B), and Seb1 (C) around the PAS, averaged from two biological replicas. Protein-coding genes for analysis were selected as above (n = 1,735). (D) Pcf11, Rhn1, and Seb1 profiles on individual genes. (E) Binding of SUMO-Pcf11-CID to the FAM-tagged two-repeat non-phosphorylated or phosphorylated CTD peptides measured by FA. Error bars show the SD of two technical repeats. (F) FA assay for Seb1-CID. Error bars show the SD of two technical repeats. See also Figures S3 and S4 and Table S1.

Since termination factors are known CTD interactors, we tested whether the termination defect in *dis2*Δ was caused by impaired recruitment of these factors. Our analysis revealed a modest, but significant increase in the recruitment of Pcf11 and Rhn1 upon Dis2 deletion (Figures 4A, 4B, 4D, S3A, S3B, S3D, and S3E, two-sided Wilcoxon rank-sum test p value < 0.001). Surprisingly, Seb1 recruitment to chromatin (Figures 4C, 4D, S3C, and S3F, two-sided Wilcoxon rank-sum test p value <0.001) and association with Pol II (Figure S3G, lanes 3 and 4) were oppositely affected in *dis2*Δ. At the same time, levels of Seb1 in the whole-cell extract were not affected in *dis2*Δ suggesting that co-transcriptional loss of Seb1 was not a result of the decreased cellular levels of Seb1 (Figure S3G, lanes 1 and 2).

To test whether termination factors directly interact with Thr4P, we purified recombinant *S. pombe* Seb1 and Pcf11 CID (Figure S4A) and tested their binding to 5′-fluorescein amidite (FAM)-labeled, differently modified two-repeat CTD peptides using fluorescence anisotropy (FA) (Figures 4E and 4F). This experiment showed that Pcf11 has similar affinity to Ser2P and Thr4P peptides (Table S1) in support of Pcf11 interacting with Ser2P and Thr4P Pol II. In agreement with our previous data (Wittmann et al., 2017), Seb1 showed preference to Ser2P, however, lower affinity for Thr4P peptides (Table S1). Since both these phospho-marks are enriched at the 3′ end of genes, we tested whether Seb1 binds to the double phospho-peptide, which is indeed the case with an affinity close to the value for the Thr4P peptide (Figure S4B). These data suggest that the loss of Seb1 observed *in vivo* could be at least in part affected by increased Thr4P in *dis2*Δ. It is also possible that Seb1 loss is mediated via increased levels of Pcf11 and Rtt103 leading to change in the stoichiometry of CTD interaction with Seb1. However, considering that affinities are not high for both proteins it is possible that other factors contribute to the loss of Seb1 in the absence of PP1 activity. We also investigated affinities to phosphorylated CTD peptides for *S. cerevisiae* termination factors: Pcf11, Rtt103, and Nrd1 (Figures S4C–S4F). All these termination factors can bind Thr4P *in vitro* (Table S1). In agreement with recent studies (Harlen et al., 2016, Jasnovidova et al., 2017, Nemec et al., 2017), Rtt103 (Rhn1 in *S. pombe*) interacts equally well with Ser2P and Thr4P (Figure S4D; Table S1). All proteins show no binding to Tyr1P in support of the proposed role for this residue in negatively affecting recruitment of termination factors (Mayer et al., 2012).

### Structural Analysis of Pol II-Seb1 Interactions

**Figure 5:**
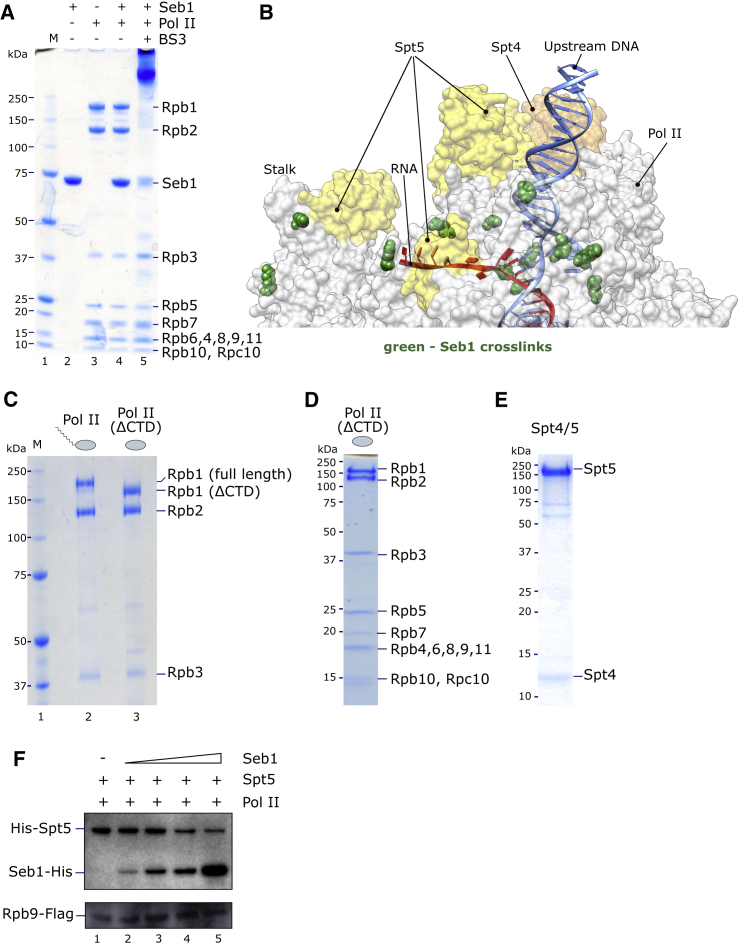
Seb1 Competes with Spt5 for Binding to Pol II (A) SDS-PAGE analysis of the purified recombinant Seb1, Pol II, reconstituted Pol II-Seb1 complex, and Pol II-Seb1 complex cross-linked with BS3. (B) Pol II crystal structure with Spt4/5 (PDB ID: 5XON) showing mapped positions of lysine-lysine cross-links (green spheres) between fission yeast Pol II subunits and Seb1. (C) SDS-PAGE analysis of Pol II before and after tobacco etch virus (TEV) cleavage. (D) Ion-exchange purification of CTD-less Pol II. (E) SDS-PAGE analysis of the purified recombinant Spt4/5. (F) The Pol II-Spt4/5 complex was incubated with increasing amount of recombinant purified Seb1. The competition between Seb1 and Spt5 for Pol II binding was analyzed by western blot with antibodies specific for Seb1 (His), Spt5 (His), and Pol II subunit Rpb9 (Flag). See also Figure S5 and Table S2.

To investigate further how Seb1 interacts with Pol II, we subjected a Pol II-Seb1 complex to cross-linking coupled to mass spectrometry analysis (Figure 5A). Several independent measurements revealed more than 150 unique (1,904 total) high-confidence lysine-lysine cross-links (1) within Pol II, (2) within Seb1, and (3) between Seb1 and Pol II (Table S2). Unique cross-links observed within the Pol II complex are in good agreement with high resolution structural data (Bernecky et al., 2016, Cramer et al., 2001, Ehara et al., 2017), where the majority of cross-links mapped on Pol II is within a 30-Å distance between Cα atoms of recovered peptides (Figure S5A). The long-distance cross-links within the Pol II complex are predominantly found between the flexible clamp of Rpb1 and Rpb2 subunits, which could reflect either extensive structural rearrangements or different populations of structural conformations. Additionally, all unique cross-links found in Seb1 RNA recognition motif (RRM) and CID domains (Table S2) are also well supported by published structural data (Wittmann et al., 2017). Strikingly, our analysis revealed cross-links between Seb1 RRM and RRM-proximal regions and several Pol II subunits: Rpb1, Rpb2, Rpb4, Rpb7, Rpb3, Rpb5, and Rpc10 (Figure S5B). Interestingly, in agreement with our data, interaction between Seb1 and Rpb7 was previously proposed based on two-hybrid screen (Mitsuzawa et al., 2003). Since there are no lysines in Pol II CTD, no cross-links were detected between Seb1 CID and the CTD. Based on our data, we conclude that, in addition to Rpb1 CTD, Seb1 forms extensive contacts with Pol II including regions near the RNA exit channel and Rpb4/7 stalk.

### Seb1 Competes with Spt5 for Binding to Pol II

The cross-links between Seb1 RRM and Pol II are found in proximity to the regions of the Pol II core where elongation factor Spt4/5 has been shown to contact (Figures 5B and S5B) (Ehara et al., 2017). This raises the exciting possibility that Spt5 and Seb1 might compete for Pol II binding at the end of the transcription cycle. To test this hypothesis, we generated a strain where CTD can be cleaved off to purify CTD-less Pol II (Figures 5C and 5D). We also purified full-length recombinant Spt4/5 heterodimer (Figure 5E) and reconstituted the Pol II (ΔCTD)-Spt4/5 complex *in vitro* (Figure 5F, lane 1). Interestingly, addition of increasing amounts of Seb1 to the Pol II (ΔCTD)-Spt4/5 complex reduced the amount of Spt5 on Pol II, suggesting that Seb1 and Spt5 compete for binding to Pol II (Figure 5F, lanes 2–5).

### Dis2 Regulates Seb1 Recruitment by Dephosphorylating Spt5

**Figure 6:**
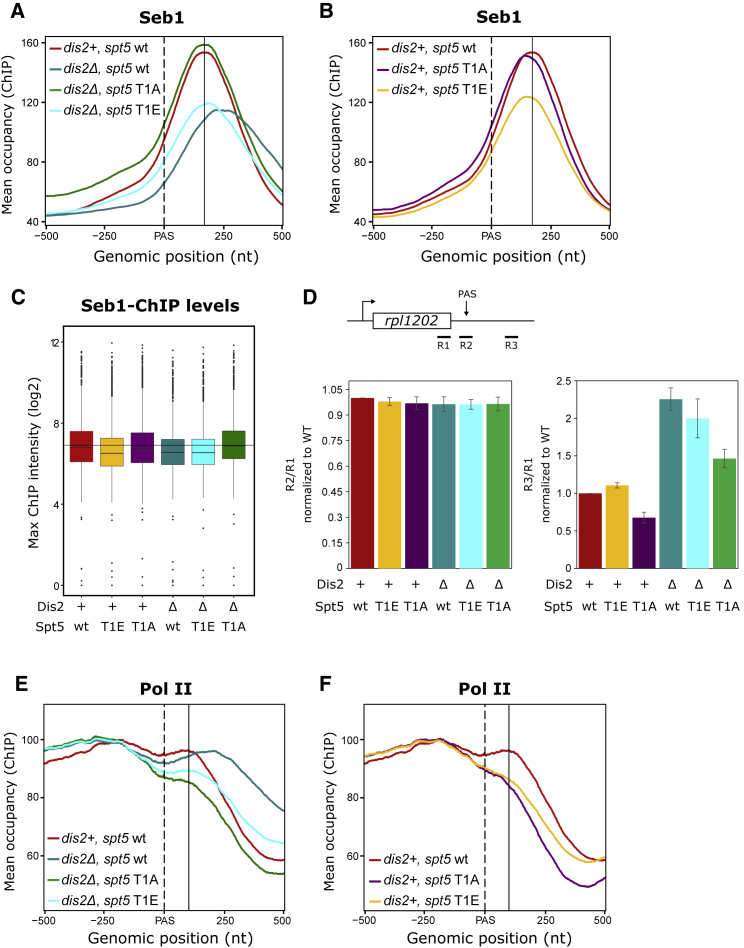
Spt5 T1A Rescues Seb1 Recruitment (A and B) Spike-in normalized ChIP-seq meta-profiles of Seb1 around the PAS in *dis2^+^, spt5* wt and *dis2Δ*, *spt5* wt/T1A/T1E (A) or *dis2^+^*, *spt5* wt/T1A/T1E (B) from four biological replicas (*dis2*^*+*^, *spt5* wt/T1E and *dis2Δ*, *spt5* wt/T1A/T1E) or three biological replicas (*dis2*^*+*^, *spt5* T1A). Protein-coding genes for analysis were selected as above (n = 1,735). The black solid line defines the position of ChIP-seq peak in the WT (*dis2*^+^*spt5* wt) strain. (C) Quantitation (boxplots) of Seb1 ChIP signal from (A) and (B). (D) Analysis of termination defects on the *rpl1202* gene. Position of the RT-PCR products is shown in the schematic. Data are represented as mean ± SEM. (E and F) Spike-in normalized ChIP-seq meta-profiles of total Pol II (8WG16) around the PAS in *dis2^+^, spt5* wt and *dis2Δ*, *spt5* wt/T1A/T1E (E) or *dis2^+^*, *spt5* wt/T1A/T1E (F) averaged from two biological replicas. Protein-coding genes for analysis were selected as above (n = 1,735). The normalized profiles were horizontally translated to have approximately equal occupancy levels upstream of the PAS. The black solid line defines the position of ChIP-seq peak in the WT (*dis2*^*+*^*, spt5* wt) strain. See also Figure S6.

Recent studies demonstrated that Spt5 stays associated with chromatin until Pol II transcribes through the PAS (Baejen et al., 2017, Shetty et al., 2017). At the end of transcription, Spt5 is dephosphorylated by Dis2 (Parua et al., 2018), which could affect the ability of Spt5 to interact with Pol II or Seb1. It is therefore possible that Dis2 facilitates termination of Pol II by mediating an interplay between elongation (Spt5) and termination (Seb1) factors during PAS transition. To investigate the relationship between Spt5 and Seb1 at the end of transcription, we replaced Thr1 on all the repeats harboring this residue (17 out of the 18) within the Spt5 CTR with either an alanine (T1A) or a phosphomimetic glutamic acid (T1E). Notably, all *spt5* mutants were stable at the protein level with amounts comparable to the WT Spt5 (Figure S6A). We then analyzed the effect of these mutations on Seb1 recruitment by ChIP-seq. Similar to our results with the *dis2* mutant, Seb1 occupancy was strongly reduced in *spt5* T1E and *spt5* T1E *dis2* mutants (Figures 6A–6C). Reciprocally, levels of Seb1 reverted to WT when phosphorylation of Spt5 was prevented in T1A (Figures 6A and 6C). This implies that Spt5 phosphorylation controls Seb1 recruitment during late stages of transcription and loss of Seb1 in the *dis2* mutant is primarily due to hyper-phosphorylation of Spt5.

### Dis2 Contributes to Pol II Termination by Dephosphorylation of Spt5 and Pol II CTD

**Figure 7:**
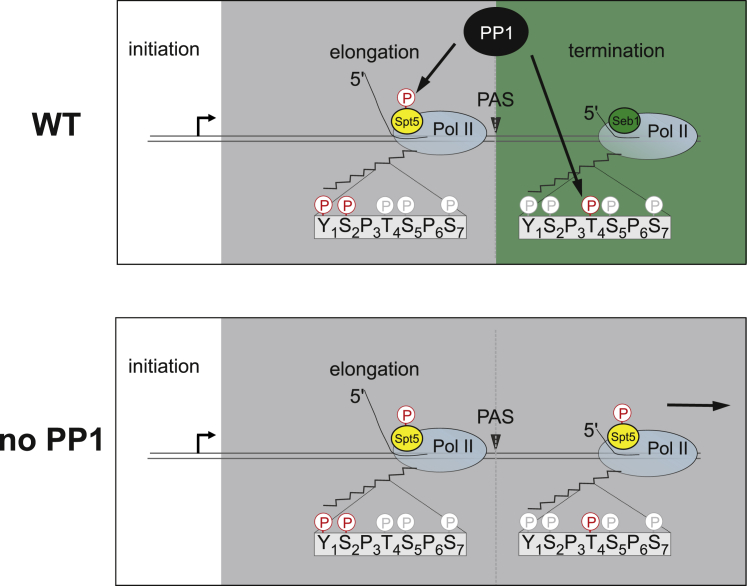
Model PP1-Dis2 dephosphorylates Spt5 and CTD-Thr4P at the end of the transcription cycle (WT). Upon depletion of PP1, hyper-phosphorylation of both Spt5 and Thr4P causes defects in transcription termination (no PP1).

Next, we wanted to investigate whether reduced Seb1 recruitment in the *dis2* mutant is the reason for delayed termination of Pol II transcription in this strain. We therefore compared levels of the 3′-extended readthrough RNA from *rpl1202* in *dis2Δ* single and *spt5* T1A *dis2Δ* double mutants where Seb1 recruitment was reversed back to WT levels (Figure 6A). We performed RT-PCR using either primer downstream or spanning the PAS (Figure 6D). As predicted, the downstream primers detected accumulation of the 3′-extended readthrough RNA in *dis2Δ*, whereas the intensity of PCR bands using primers spanning the PAS was the same in both *dis2Δ* and control strain. Based on these observations, we conclude that Dis2 regulates transcription termination after completion of the mRNA cleavage. We reasoned that if the termination defect is only due to the reduction in Seb1 recruitment, we should observe suppression of the termination defect when Seb1 levels go back to normal in this strain. Surprisingly, only partial suppression of the termination defect is observed (Figure 6D), suggesting that, although reduced Seb1 recruitment is partly responsible, it is not the sole reason for the delayed release of Pol II in *dis2*Δ. On the other hand, loss of Seb1 due to mutated Spt5 in the presence of functional Dis2 (Figure 6B) was not sufficient to cause accumulation of the readthrough (Figure 6D). Consistent with the RT-PCR data, our global analysis of Pol II occupancy also revealed partial rescue of the termination defect upon loss of Spt5 phosphorylation (T1A mutant) in *dis2Δ* strain (Figures 6E and S6B). At the same time, introducing phosphomimetic mutation in Spt5 (*spt5* T1E) did not lead to delayed Pol II termination in the presence of active Dis2 (Figures 6F and S6B) and correlates with lack of the 3′ readthrough observed in this strain (Figure 6D). This is in contrast to what is observed in *dis2* mutant (Figures 6E and S6B). We therefore conclude that the termination defect seen in *dis2*Δ is due to hyper-phosphorylation of both Dis2 substrates: Spt5 and Thr4P in the Pol II CTD (Figure 7).

## Discussion

During the transcription cycle, Pol II progresses through three main stages: initiation, elongation, and termination. Different sets of factors interact with the Pol II core as well as with the phosphorylated CTD of Rpb1 to regulate the transition from initiation to elongation and from elongation to termination. These two transitions are associated with Pol II pausing at the beginning and at the end of genes (Guo and Price, 2013, Kwak and Lis, 2013, Laitem et al., 2015, Yamaguchi et al., 2013). Interestingly, recent studies have demonstrated that peaks of paused Pol II are also characterized by enrichment of specific CTD phospho-isoforms in mammals (Laitem et al., 2015, Nojima et al., 2015, Schlackow et al., 2017). Here, we report the first systematic analysis of the distribution of Pol II phospho-marks in fission yeast. We find pausing of Pol II at the 3′ end of genes, in agreement with previous work (Booth et al., 2016). The 3′ paused Pol II is phosphorylated at Ser2, Tyr1, and Thr4, resembling the patterns observed in mammalian cells. Unlike mammals, fission yeast does not show widespread promoter-proximal Pol II pausing nor enrichment of Tyr1P at the 5′ end of genes. The co-occurrence of multiple phospho-marks at gene 3′ ends is likely to reflect the complexity of events that take place at this stage of the transcription cycle, when multiple proteins are recruited to form dynamic complexes required for correct mRNA processing, mRNA release, and Pol II dissociation from the DNA template. Due to the complex nature of this process, the current knowledge of the mechanisms involved in transcription termination is very limited.

What is the function of Thr4P at the gene 3′ ends? Here, we show that substitution of all threonines to alanines (T4A) within the canonical repeats of CTD does not significantly affect cell growth in fission yeast. This is consistent with work in budding yeast, where T4A and T4V mutants are also viable (Harlen et al., 2016, Nemec et al., 2017). Moreover, recent studies have found that *S. cerevisiae* Rtt103 can bind equally well Ser2P and Thr4P (Harlen et al., 2016, Jasnovidova et al., 2017, Nemec et al., 2017). Here, we show that the two essential CID-containing termination factors in *S.pombe,* Pcf11 and Seb1, also bind Thr4P. Despite the emerging importance of Thr4P, very little is known about the kinases and phosphatases that regulate this phospho-mark. We identify Dis2 as a Pol II CTD phosphatase that acts at the gene 3′ ends with specificity for Thr4P and demonstrate that Dis2 is important for Pol II termination genome-wide. Moreover, we report that substitution of threonine 4 to phosphomimetic glutamic acid (T4E) in all canonical repeats of CTD leads to cell death, suggesting that removing Thr4P is critical. Since loss of Dis2 does not lead to lethality, the T4E mutated CTD is likely to also affect elongation. Indeed, high levels of Thr4P within the gene body of long non-coding transcripts have been shown to correlate with abortive transcription in mammals (Schlackow et al., 2017). We show that CID factors bind to Thr4P suggesting that their precocious tethering during transcription by increased Thr4P can be a reason for premature termination. On protein-coding genes Thr4P is depleted from gene body. This can be either due to the recruitment of Thr4P kinase specifically at the end of the transcription cycle, or, alternatively, there is another phosphatase that acts during transcription elongation. Interestingly, Dis2 ortholog, Glc7, also targets the CTD. However, Glc7 is important for dephosphorylating Tyr1P (Schreieck et al., 2014) and therefore is likely to act via a mechanism distinct from Dis2.

Although the mechanism underlying Pol II release from DNA is largely unknown, we previously demonstrated a key role for the CTD- and RNA-binding factor Seb1 in mRNA 3′ processing and transcription termination (Wittmann et al., 2017). Here, we show that Seb1 forms extensive contacts with the Pol II core via its RRM. Recent structural studies of Pol II in complex with initiation and elongation factors have revealed that the enzyme’s properties can be modulated by binding of transcription factors to the Pol II core (Ehara et al., 2017, Glyde et al., 2017, Plaschka et al., 2015, Schulz et al., 2016, Xu et al., 2017). It was proposed that initiation factors control the conformational state of the clamp as well as the catalytic properties of Pol II in a different way than elongation factors do (Schulz et al., 2016). We speculate that Seb1 interaction with the Pol II core might modulate Pol II properties to facilitate its dislodgement from DNA. We envision that binding on Pol II near the RNA exit channel would enable Seb1 to interact with its binding site on nascent RNA as it emerges from the channel. RNA binding is essential for Seb1 recruitment, correct mRNA processing, and transcription termination (Wittmann et al., 2017). Binding to phosphorylated CTD is also essential for Seb1 function, although not for its recruitment (Wittmann et al., 2017). It is possible that phosphorylated CTD allosterically regulates Seb1 and its interactions with the Pol II core or CPF. Failure to timely terminate transcription observed in the PP1 mutant and lethality of the CTD T4E mutant further support the importance of CTD phosphorylation for proper transcription termination.

Interestingly, we observe that Seb1 contact sites on Pol II overlap with the surface occupied by Spt5. Spt5 regulates the transition from initiation to elongation by (1) displacing the initiation factor TFIIE from Pol II (Grohmann et al., 2011, Schulz et al., 2016), (2) mediating promoter-proximal pausing via recruitment of the negative elongation factor (NELF), (3) releasing the paused Pol II (Missra and Gilmour, 2010). Spt4/Spt5P is proposed to stimulate the processivity of Pol II by preventing its dissociation from DNA and stabilizing the Pol II elongation complex (Werner, 2012). Spt5 stays associated with chromatin until Pol II transcribes past the PAS, at which point it binds to the nascent RNA (Baejen et al., 2017, Shetty et al., 2017). Interestingly, our data show Seb1 and Spt5 compete for binding to Pol II *in vitro*, suggesting that, at the transition from elongation to termination, Spt5 needs to dissociate from the Pol II core for Seb1 to bind and termination to occur. Interestingly, we demonstrate an antagonistic relationship between Spt5 and Seb1 that depends on Spt5 phosphorylation and is regulated by Dis2. There could be several possible explanations for the role of Spt5 phosphorylation at the 3′ end of protein-coding genes. (1) Spt5 phosphorylation prevents its dissociation from Pol II and blocks Seb1 recruitment, thus leading to impaired transcription termination in *dis2*Δ. However, Spt5 was shown to form a complex with Pol II in its unphosphorylated form early during transcription (Peterlin and Price, 2006, Renner et al., 2001, Wu et al., 2003, Yamaguchi et al., 1999) arguing against this scenario. (2) Phosphorylated Spt5 bound to Pol II creates a negatively charged surface that interferes with Seb1 binding to Pol II. (3) Another possibility is that non-phosphorylated Spt5 directly recruits Seb1, as it was proposed for the guanylyltransferase and triphosphatase modules (Pce1 and Pct1) of the capping enzyme (Doamekpor et al., 2014, Doamekpor et al., 2015, Schneider et al., 2010) and the NELF (Missra and Gilmour, 2010). However, our *in vitro* data suggest that two proteins are unlikely to co-occupy the same molecule of Pol II.

Unlike other Pol II interacting factors, Spt5 is universally conserved in archaea and bacteria (NusG) reflecting its importance in transcription regulation. Interestingly, a possible link between NusG and termination factor Rho has been suggested in bacteria (Cardinale et al., 2008, Dutta et al., 2008, Peters et al., 2012). Although the nature of NusG-Rho relationship is not well understood, it is tempting to speculate that communication between elongation and termination factors to regulate properties of RNA polymerase is conserved across different domains of life.

Our study provides an important insight into the understanding of fundamental principles underlying Pol II release from DNA by demonstrating that, similar to general initiation and elongation factors, the termination factor Seb1 forms extensive interactions with the Pol II core. Moreover, we demonstrate an important function of the PP1 phosphatase Dis2 in orchestrating the transition between elongation and termination by dephosphorylating Spt5 and Pol II CTD. Based on our data, the following model is emerging: Spt5 and its phosphorylation regulate two major transitions that Pol II undergoes at the beginning and at the end of transcription cycle (initiation to elongation and elongation to termination) (Figure 7). During these transitions different sets of transcription factors interact with Pol II in a Spt5-dependent manner and modulate properties of the transcribing Pol II enabling stage-specific functions. Future biochemical and structural studies should help to test this model and further dissect the mechanism of termination.

## STAR★Methods

### Key Resources Table

**Table:** 

REAGENT or RESOURCE	SOURCE	IDENTIFIER
**Antibodies**		

anti-Pol II (8WG16) (mouse)	Millipore	Cat#05-952; RRID:AB_492629
anti-Pol II CTD phospho Ser2, Clone 3E10 (rat)	Millipore	Cat#04-1571-I; RRID:AB_11212363
anti-Pol II CTD phospho Ser5, Clone 3E8 (rat)	Millipore	Cat#04-1572-I; RRID:AB_10615822
anti-Pol II CTD phospho Ser7, Clone 4E12 (rat)	Active Motif	Cat#61087; RRID:AB_2687452
anti-Pol II CTD phospho Tyr1, Clone 3D12 (rat)	Active Motif	Cat#61383
anti-Pol II CTD phospho Thr4, Clone 6D7 (rat IgG2b)	Active Motif	Cat#61361
anti-Flag-HRP antibody	Sigma Aldrich	Cat#A8592; RRID:AB_439702
anti-His-HRP antibody	Clontech	Cat#631210
Anti-tubulin antibody (rat)	Abcam	Cat#ab6160: RRID:AB_305328

**Chemicals, Peptides, and Recombinant Proteins**		

Anti-Flag M2 affinity gel	Sigma-Aldrich	Cat#A2220; RRID:AB_10063035
3xFlag peptide	Sigma-Aldrich	Cat#F4799
Protein G Dynabeads	ThermoFisher	Cat#10004D
Rabbit IgG Agarose	Sigma-Aldrich	Cat#A2909; RRID:AB_1172450
Agencourt AMPure XP	BeckmanCoulter	Cat#A63881

**Critical Commercial Assays**		

ChIP DNA Clean & Concentrator Kits	ZymoResearch	Cat#D5205
NEBNext Ultra DNA library Kit for Illumina	NEB	Cat#E73705
NEBNext Fast DNA Library Prep Set for Ion Torrent	NEB	Cat#E62705

**Deposited Data**		

ChIP-seq	This study	GEO: GSE111326

**Experimental Models: Organisms/Strains**		

*S.pombe* strains	This study and other sources	See Table S3
*S.cerevisiae* strains	Other sources	See Table S3

**Oligonucleotides**		

Rpl1202_gb2_F:GTCAGATATGCAAGAGAAACCTA	This study	N/A
Rpl1202_gb2_R:GTAAACGGATTATGTGCTTGC	This study	N/A
Rpl1202_rt1_F:AAACCCGACATCAACAACAA	This study	N/A
Rpl1202_rt2_F:GCTTACAACGATTATG AACTTAACAGG	This study	N/A
Rpl1202_rt2_R:ATGTTCATACTTGATGAATGGTCC	This study	N/A

**Recombinant DNA**		

pET41a(+)-SpSeb1-CID(1-148)-His8	This study	N/A
pET41a(+)-ScPcf11-CID(1-144)-His8	This study	N/A
pET41a(+)-ScRtt103-CID(1-162)-His8	This study	N/A
pET41a(+)-ScNrd1-CID(1-152)-His8	This study	N/A
pOPINS3C-SUMO-His6-SpPcf11-CID(1-139)	This study	N/A
pRSFDuet-His8-Spt5wt-Spt4	This study	N/A
pET41a(+)-Seb1(full-length)-His8	(Wittmann et al., 2017)	N/A

**Software and Algorithms**		

Bowtie2	(Langmead and Salzberg, 2012)	N/A
Samtools	(Li et al., 2009)	N/A
Deeptools	(Ramírez et al., 2016)	N/A
GeneSelection.R	This study	https://github.com/Kec89/Gene-selection-

### Contact for Reagent and Resource Sharing

Further information and requests for resources and reagents should be directed to and will be fulfilled by the Lead Contact, Lidia Vasiljeva (lidia.vasilieva@bioch.ox.ac.uk).

### Experimental Model and Subject Details

#### Yeast strains and manipulation

*S. pombe* strains were grown in YES medium at 30°C to OD_600_ of 0.4 - 0.7 before harvesting. Standard PCR-based methodology was used for epitope tagging (Bähler et al., 1998). Strains used in this study are listed in Table S3.

### Method Details

#### Construction of Spt5-CTR and Rpb1-CTD point mutants

*S. pombe* Spt5-CTR sequences of Thr1 mutants (T1A and T1E) with an optimized codon usage were synthesized by Eurofins Genomics. 17 out of the 18 repeats within the CTR have a Thr1 at position one and the T1A/T1E mutations were introduced in all these 17 repeats.

*S. pombe* Rpb1-CTD sequences of Thr4 mutants (T4A and T4E) with an optimized codon usage were synthesized by Eurofins Genomics. In the *S. pombe* CTD, the first four repeats deviate from the consensus heptad (YSPTSPS). T4A and T4E mutations were introduced within the distal 25 heptads that perfectly match to the consensus sequence (with the exception of an alanine instead of Pro3 in the ninth heptad). Rpb1 CTD T4A and T4E were transformed into diploid yeast cells. Tetrad dissection was performed following a 3 days incubation of diploid strains on sporulation medium (EMMG) at 30°C.

#### ChIP-seq

All ChIP-seq experiments were performed in at least duplicates. Chromatin was prepared as previously described (Wittmann et al., 2017). For spike-in control, *S. cerevisiae* cells were added to *S. pombe* cells before cross-linking. *S. cerevisiae* cells correspond to 15% of *S. pombe* cells. Immunoprecipitations were conducted with IgG agarose beads (Sigma) or antibodies against Rpb1 (8WG16, Millipore), Tyr1P-CTD (3D12, ActiveMotif), Ser2P-CTD (3E10, Millipore), Thr4P-CTD (6D7, ActiveMotif) coupled to protein G dynabeads (Life Technologies) as described in (Wittmann et al., 2017) except that when immunoprecipitating with Thr4P antibody, NaCl was used at concentration of 150 mM in all buffers. Libraries were constructed using NEBNext Fast DNA Library Prep Set for Ion TorrentTM Kit or NEBNext Ultra DNA Library Prep Kit for Illumina. Libraries were sequenced on the Ion Torrent Proton or the Illumina NextSeq500.

#### ChIP-seq data analysis

Reads were aligned to a concatenated genome (*S. pombe* + *S. cerevisiae*) using Bowtie2 (Langmead and Salzberg, 2012). Reads that mapped more than once were discarded and PCR duplicates were removed using SAMtools (Li et al., 2009). To assess changes in protein occupancy between strains, a spike-in normalization was used where *S. pombe* reads were adjusted using the number of *S. cerevisiae* reads in each IP sample. Genomic DNA sequencing of the input mixture of fission and budding yeast was also used to correct for any variation in cell mixture ratios. For metagene profiles and single gene analysis, *S. pombe* genome annotation from (Eser et al., 2016) was used. The analysis was done over a subset of 1735 protein-coding genes unless otherwise stated. These 1735 genes have a distance greater than 275 nt from the TSS of the downstream gene (on the same strand) and do not overlap with any other transcription unit (on the opposite strand) in a region from 250 nt upstream to 500 nt downstream of the PAS.

#### Pol II termination index

Pol II termination index was determined by dividing mapped reads in window B1 or B2 by the read count within [PAS ± 500 nt]. B1 corresponds to the region from PAS to PAS+50 nt. B2 corresponds to the region from PAS+350 nt to PAS+400 nt. For each strain, the relative value for B2 was calculated by setting the median of B1 arbitrarily to 1.

#### PP1 phosphatase assay

Flagged-Dis2 *S. pombe* strain (1L) was grown at 30°C and harvested at the exponential phase, frozen in liquid nitrogen and cells were disrupted by manual grinding. Cells were mixed with the lysis buffer (50 mM Tris-HCl pH 7.5, 150 mM NaCl, 10% glycerol, 0.5% Triton X-100, 0.5 mM DTT, 0.5 mM MgCl_2_, 0.5 mM Mg(OAc)_2_) containing a proteinase inhibitor cocktail. Lysate was clarified by centrifugation at 16000 g for 20 minutes and incubated with 100 μl of M2 agarose gel (Sigma) for 2 hours. Beads were washed 4 times with lysis buffer, 4 times with buffer A (lysis buffer without Triton X-100), then Dis2 complexes were eluted with four volumes of 2.5 mg/ml Flag-peptide (Sigma) in buffer B (25 mM Tris-HCl pH 7.5, 150 mM NaCl, 10% glycerol, 3 mM DTT, 0.5 mM MgCl_2_, 0.5 mM Mg(OAc)_2_).

Flagged-Rpb9 Pol II (2L) was purified essentially as above. The M2 agarose gel (400 μl) was washed 4 times with buffer C (50 mM Tris-HCl pH 7.5, 1 M NaCl, 1 M urea, 10% glycerol, 0.5% Triton X-100, 0.5 mM DTT, 0.5 mM MgCl_2_, 0.5 mM Mg(OAc)_2_), 4 times with buffer A, then eluted as above.

For the dephosphorylation assay, Pol II (1L) was incubated with Dis2 (1L) at 30°C for the indicated time in buffer B (25 mM Tris-HCl pH 7.5, 150 mM NaCl, 10% glycerol, 3 mM DTT, 0.5 mM MgCl_2_, 0.5 mM Mg(OAc)_2_). In parallel, Pol II (1L) was incubated without Dis2 as a negative control. Reactions were stopped by addition of SDS-buffer and phosphorylation status of Rpb1 was assessed with phospho-specific antibodies.

#### RT-PCR

RNA was extracted as previously described (Vasiljeva and Buratowski, 2006). Reverse transcription of DNase-treated total RNA was performed using gene-specific primers followed by PCR. A control not containing reverse transcriptase (-RTase) was included. RT–PCR products were resolved on 1.2% agarose gel, and ethidium-stained PCR products were quantified with ImageJ. For each set of primers, the relative intensities of PCR products 2 and 3 were calculated by setting that of PCR product 1 arbitrarily to 1. Intensities from the -RTase control were always 0. Primers used are listed in Key Resources Table.

#### Fluorescent anisotropy (FA) assay

The *S. cerevisiae* Nrd1 CID1-152, Pcf11 CID1-144, Rtt103 CID1-152 and the *S. pombe* Seb1 CID1-148 constructs were cloned into pET41a(+), resulting in a C-terminal His(x8)-tag. The *S. pombe* Pcf11 CID1-139 with an N-terminal His6-SUMO tag was expressed from modified pOPINS3C expression vector (Bird, 2011). *S.c*. Pcf11 was expressed and purified as in (Mayer et al., 2012). Nrd1, Rtt103 and Seb1 were purified as in (Wittmann et al., 2017). Briefly, after elution from Ni-NTA agarose beads (QIAGEN), protein-containing fractions were pooled and subjected to size-exclusion chromatography using a HiLoad 16/60 Superdex 200 prep grade column (GE Healthcare) on an ÄKTA purifier (GE Healthcare). *S. pombe* Pcf11 was subjected to size-exclusion chromatography using a HiLoad 16/60 Superdex 75 prep grade column (GE Healthcare) to eliminate soluble but high molecular species. For *S. cerevisiae* Pcf11, Ni-NTA elution fractions were loaded on a MonoQ anion exchange column (Amersham Bioscience) prior to size-exclusion chromatography

For FA assays, binding to 75 nM of two-repeat CTD peptides containing an N-terminal 5′-fluorescein amidite (FAM)-tag (Peptides&elephants, Potsdam, Germany) was determined after incubation at 25 °C for 20 min. For *S.c.* Rtt103, *S.c*.Nrd1 and *S.p*. Pcf11 measurements were performed in the presence of 150 mM NaCl. For *S.p.* Seb1 measurements were done in the presence of 200 mM NaCl, and for *S.c*. Pcf11 in the presence of 50 mM NaCl. Excitation of the ligand was performed with linearly polarized light at 485 nm and emission was measured both parallel and perpendicular to plane at 520 nm at 25 °C using a FLUOstar-Omega microplate reader (BMG-Labtech). Results were plotted against the protein concentration and Kd values were determined via curve fitting as described in (Reinstein et al., 1990).

#### Pol II and Seb1 cross-linking

Large-scale *S. pombe* Pol II purification was performed as above with the following modifications. Cells were disrupted in a freezer mill (SPEX SamplePrep). The cell lysate was incubated with M2 beads for 1.5 h. Protein was eluted with 5 mL of 5 mg/ml Flag-peptide (Sigma), followed by addition of 20 mL of QA buffer (50 mM TRIS pH 7.7, 5 mM NaCl, 10% glycerol, 0.5 mM MgCl_2_, 0.5 Mg(OAc)_2_, 1 mM β-mercaptoethanol). Protein eluate was then applied to an ion exchange chromatography column (2x 1ml HiTrap Q HP, GE Healthcare) equilibrated with QA buffer. The column was washed with several column volumes of 8% buffer QB (same as QA, except 2000 mM NaCl) until a stable baseline was achieved. Protein was eluted with a gradient of QB buffer (up to 40%). Fractions containing Pol II were mixed with 75% glycerol to reach 50% and stored at −20°C until the day of the experiment. For cross-linking, Pol II buffer was exchanged to CB buffer (50mM HEPES pH 7.6, 150 mM NaCl, 5% glycerol, 1mM DTT, 0.5 mM MgCl_2_ and 0.5 mM Mg(OAc)_2_) and concentrated with Vivaspin 50 kDa MWCO (GE Healthcare).

Full-length Seb1 was expressed as a fusion protein with C-terminal His8-tag (from pET41a plasmid) in Rosetta *E. coli* strain, grown at 37°C and induced with 0.5 mM IPTG for 4h. Cells were collected by centrifugation at 4 °C and 5000 g for 20 min. Frozen pellets were re-suspended in NA buffer (50 mM Tris-HCl pH 7.7, 600 mM NaCl, 5 mM imidazole, 1 mM β-mercaptoethanol) supplemented with protein inhibitor cocktail, followed by lysis in French Press and addition of phenylmethylsulfonyl fluoride (PMSF) to 1 mM. Lysates were cleared at 4 °C, 20000 g for 30 min, filtered and loaded onto a nickel-nitrilotriacetic acid resin (QIAGEN, 1ml slurry per 2l of culture) and incubated for 1h. Protein was eluted with 500 mM imidazole. Fractions containing protein were centrifuged at 10000 g for 10 min and separated on Superdex 200 10/300 GL (GE Healthcare) equilibrated in CB buffer. Fractions containing monomeric Seb1 were concentrated (if necessary), snap-frozen in liquid nitrogen and stored at −80°C.

Complex formation between Seb1 and Pol II was assessed by pull-down experiments or western blot (for cross-linking) with anti-His and anti-Flag antibodies (data not shown).

Two cross-linking protocols were used to assess the interaction between Seb1 and Pol II.

PROTOCOL A.14 μg of Seb1:Pol II complex (6:1 molar ratio) was incubated in CB buffer with 60 μg of BS3 for 30 min at 4 °C. The reaction was then quenched by boiling in Laemmli buffer. Samples were resolved on NuPAGE 4%–12% Bis-Tris Gels (ThermoFisher Scientific). The cross-linked band was cut and subjected to in-gel trypsin digestion as previously described (Shevchenko et al., 2006). After digestion, peptides were extracted using acetonitrile 80% (v/v), Trifluoroacetic acid 0.1% (v/v), dried down in SpeedVac Concentrator (Thermo Fisher Scientific), and reconstituted in formic acid 5% (v/v), dimethyl sulfoxide 5% (v/v) for MS analysis.

PROTOCOL B. For enrichment of cross-linked peptides, a total of 70 μg Seb1 and Pol II (4:1 molar ratio) was cross-linked with 200 μg of BS3, the reaction was quenched with 2 μl of 1 M Tris pH 7.5. The sample was prepared following Schmidt and Robinson (2014) with a few modifications. Briefly, the sample was dried under vacuum and reconstituted in 1% Rapigest (Waters) in 25 mM ammonium bicarbonate. The reconstituted sample was reduced using 25 mM dithiothreitol for 1h at 37°C, alkylated with 33 mM iodoacetamide for 1h at 37°C, and digested with 3.5 μg of trypsin (Promega) for 14 h at 37°C. Digestion was terminated and Rapigest removed by addition of trifluoroacetic acid to a final concentration of 1% and incubation for 2h at 37°C. A thermomixer was used in all steps set at 500 rpm mixing speed. The sample was then centrifuged at 16,200 g and 4°C for 30 min, the supernatant collected and dried under vacuum for following strong cation exchange (SCX) enrichment. Trypsin-digested peptides were applied to SCX mini spin columns (Thermo Fisher) and 11 fractions (flow through, wash, 25, 50, 100, 200, 300, 400, 500, 1000 and 2000 mM ammonium acetate elutions) were analyzed by mass spectrometry.

#### Analysis of cross-linked peptides by mass spectrometry

Obtained peptides were separated by nano-flow reversed-phase liquid chromatography coupled to Q Exactive Hybrid Quadrupole-Orbitrap mass spectrometer (Thermo Fisher Scientific). Peptides were loaded on a C18 PepMap100 pre-column (inner diameter 300 μm × 5mm, 3 μm C18 beads; Thermo Fisher Scientific) and separated on an in-house packed analytical column (75 μm inner diameter x 50cm packed with ReproSil-Pur 120 C18-AQ, 1.9 μm, 120 Å, Dr. Maisch GmbH). Separation of cross-linked peptides was conducted with a first step linear gradient from 15 to 35% of B for 30 min followed by a second step from 35% to 55% of B for additional 15 min, at a flow rate of 200 nl/min (A: 0.1% formic acid, B: 0.1% formic acid in acetonitrile). All data were acquired in a data-dependent mode, automatically switching from MS to collision-induced dissociation MS/MS on the top 10 most abundant ions with a precursor scan range of 350–2000 m/z. MS spectra were acquired at a resolution of 70 000 and MS/MS scans at 17 500. Dynamic exclusion was enabled with an exclusion duration of 5 s and charge exclusion was applied to unassigned, mono- and doubly-charged ions. Raw data files were processed for protein identification using MaxQuant, version 1.5.0.35, integrated with the Andromeda search engine as described previously (Cox and Mann, 2008, Tyanova et al., 2016, Cox et al., 2011). The MS/MS spectra were searched against *S. pombe* Uniprot database, precursor mass tolerance was set to 20 ppm and MS/MS tolerance to 0.05 Da. Enzyme specificity was set to trypsin with a maximum of two missed cleavages. Protein and peptide spectral match false discovery rate was set at 0.01. For cross-linked peptides raw files were converted into mgf format using pParse and searched using the pLink software (Yang et al., 2012). In this case, the database contained the target proteins only (*S. pombe* Seb1 and Pol II subunits). Search parameters were as follows: maximum number of missed cleavages = 2, fixed modification = carbamidomethyl-Cys, variable modification 1 = Oxidation-Met, variable modification 2 = Glu to pyro-Glu, mass accuracy filter = 20 ppm for precursor ions with consideration of the first 5 isotopic peaks, MS2 tolerance = 20 ppm. Data were initially filtered by E-value (E < 1.0e-5). Cross-links were further filtered/inspected with specific emphasis on fragmentation patterns. In total 156 unique cross-linked peptides were recovered. Data was analyzed and visualized in UCSF Chimera (Pettersen et al., 2004) with Xlink Analyzer add-on (Kosinski et al., 2015) and with the online tool xiNET (Combe et al., 2015).

#### Fission yeast Pol II and Spt4/5 interaction prediction

Pol II and Spt4/5 are highly conserved among species, therefore, interaction is expected to be preserved in different organisms. A recently reported structure (Ehara et al., 2017) describing details of the interaction between Spt4/5 and Pol II of Komagataella phaffii (K. phaffii) (PDB ID: 5XON) was used to extract residues of Spt4/5 and Pol II that are in close contact (15Å cut-off). Next, alignment between Pol II subunits of *S. pombe* and *K. phaffii* were used to assign the residues of fission yeast Pol II that would interact with Spt4/5 as shown in Figure S5B. Structural alignment of Pol II structures from both species (PDB ID: 5XON for K. phaffii and 4H0G for *S. pombe*) was used to map *S. pombe* Seb1-PolII cross-links on the K. phaffii structure (Figure 5B).

#### Seb1/Spt5 competition assay for Pol II binding

A TEV cleavage site was integrated into Rpb1 upstream of the CTD. The purification of this CTD-less polymerase (Rpb9-flagged) was performed as above with the following modifications. After Flag-purification and elution, CTD was cleaved with home-made Tev protease at room temperature for 1 h. After ion exchange chromatography, the sample was subjected to size-exclusion chromatography on Superose 6 resin (GE Healthcare).

Full-length Spt4/5 was cloned into a pRSFDuet plasmid with Spt5 containing N-terminal His8-tag and thioredoxin. Expression was carried out in *Escherichia coli* BL21 strain grown to 0.8 OD at 37°C and induced with 1mM IPTG for 3h. Cells from 12L were collected by centrifugation at 6000 g, 4°C for 15 min. Pellets were resuspended in NiTA buffer (50 mM Tris-HCl pH 7.8, 500 mM NaCl, 5 mM imidazole, 1 mM 2-mercaptoethanol) supplemented with 2500 U of SuperNuclease (Sino Biological Inc.). Cells were incubated for 20 min at 4 °C, lysed by French Press at 20 kpsi and then 0.5% Tween and 1mM PMSF were added. Lysates were centrifuged at 4 °C, 30000 g for 20 min. Cleared lysed were applied to Ni-NTA resin (nickel-nitrilotriacetic acid, 1.5 mL of slurry equilibrated with NiTA, QIAGEN) and incubated for 30 min at 4 °C. The resin was washed with 5 mL of NiTA buffer and 50 mL NiTC buffer (as NiTA but 1M NaCl and 1M urea). Proteins were eluted with 200 mM imidazole and diluted with Q1 buffer (50 mM TRIS pH 7.8, 20 mM NaCl, 0.5 mM MgCl_2_, 0.5 mM Mg(OAc)_2_, 1mM 2-mercaptoethanol, 10% glycerol). Proteins were applied to ion exchange (two 1ml columns of Q HP, GE Healthcare on AKTA system). Proteins were washed with 8% of buffer Q2 (as for Q1 buffer but 2M NaCl). Proteins were eluted with a gradient of salt (up to 30% of Q2). Fractions containing Spt4/5 were concentrated and applied to gel filtration with Superdex 200 10/300 (GE Healthcare) equilibrated with GF buffer (20 mM HEPES pH 7.5, 150 mM NaCl, 0.5 mM MgCl_2_, 0.5 mM Mg(OAc)_2_, 1 mM 2-mercaptoethanol). Proteins were aliquoted and stored at −80°C until experiments.

For the competition assay, 16 ng of CTD-less Pol II (Rpb9-Flag) were immobilized on 20 μL of M2 agarose beads for 30 min at 4°C. Beads were washed 4 times with BB buffer (20 mM HEPES pH 7.5, 150 mM NaCl, 0.5% Triton X-100, 0.5 mM MgCl_2_, 0.5 mM Mg(OAc)_2_, 1 mM 2-mercaptoethanol) and resuspended in 200 μL BB buffer. Pol II was then incubated with 3 μg of Spt4/5 for 30 min at 25°C. Beads were washed 4 times with BB buffer, resuspended in 200 μL. The Pol II-Spt4/5 complex was then incubated with recombinant Seb1 (25, 75, 225 or 680 ng) for 30 min at 4°C. Beads were washed 4 times with BB buffer, then the complexes were eluted with one volume of 5 mg/ml Flag-peptide (Sigma) in buffer BB for 5min at 4°C and analyzed by western blotting.

### Quantification and Statistical Analysis

All ChIP-seq were performed in at least two biological replicas. Statistical significance of differential ChIP signal intensity was calculated by two sided Wilcoxon rank-sum test for each replicas in Supplemental Information.

### Data and Software Availability

All high throughput sequencing data used in this study have been deposited at GEO under accession number GSE111326.

All scripts written for this analysis method are available to download from https://github.com/Kec89/Gene-selection-.

## Supporting Citations

The following references appear in the Supplemental Information: Ghaemmaghami et al., 2003, Lemieux et al., 2011.

## Supplemental Information

Document S1. Figures S1–S6 and Tables S1 and S3

Table S2. Cross-Links within Pol II, within Seb1, and between Seb1 and Pol II, Related to Figures 5 and S5

Document S2. Article plus Supplemental Information
