# Supplementary material for: Elongation/Termination Factor Exchange Mediated by PP1 Phosphatase Orchestrates Transcription Termination
Source: Cell Rep. 2018 Oct 2;25(1):259–269.e5. doi: 10.1016/j.celrep.2018.09.007 (PMC6180485; doi:10.1016/j.celrep.2018.09.007)
Supplement: Document S1. Figures S1–S6 and Tables S1 and S3 [file mmc1.pdf]

Cell Reports, Volume *25*

Supplemental Information

Elongation/Termination Factor Exchange Mediated by PP1 Phosphatase Orchestrates Transcription Termination

Tea Kecman, Krzysztof Kuś, Dong-Hyuk Heo, Katie Duckett, Adrien Birot, Sabrina Liberatori, Shabaz Mohammed, Lucia Geis-Asteggiante, Carol V. Robinson, and Lidia Vasiljeva

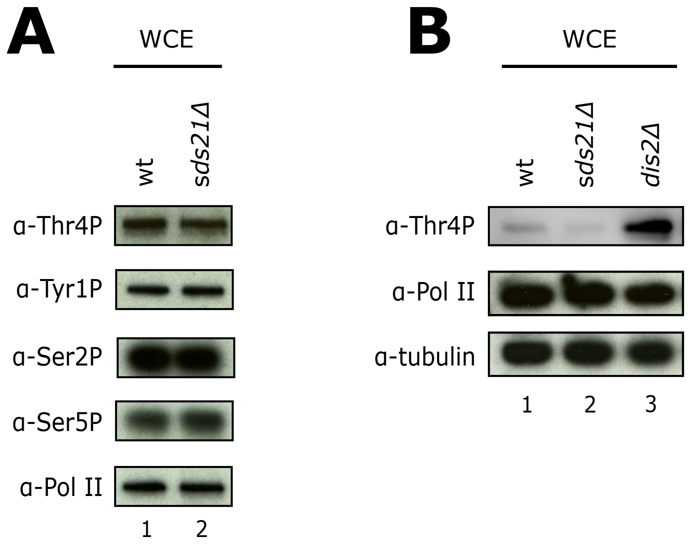

Figure S1. Sds21 is not a CTD-phosphatase, Related to Figure 2
(A-B) Whole cell extract levels of phospho-Pol II were analysed by Western blot using the indicated antibodies in wt, *sds21Δ* and *dis2Δ* strains.

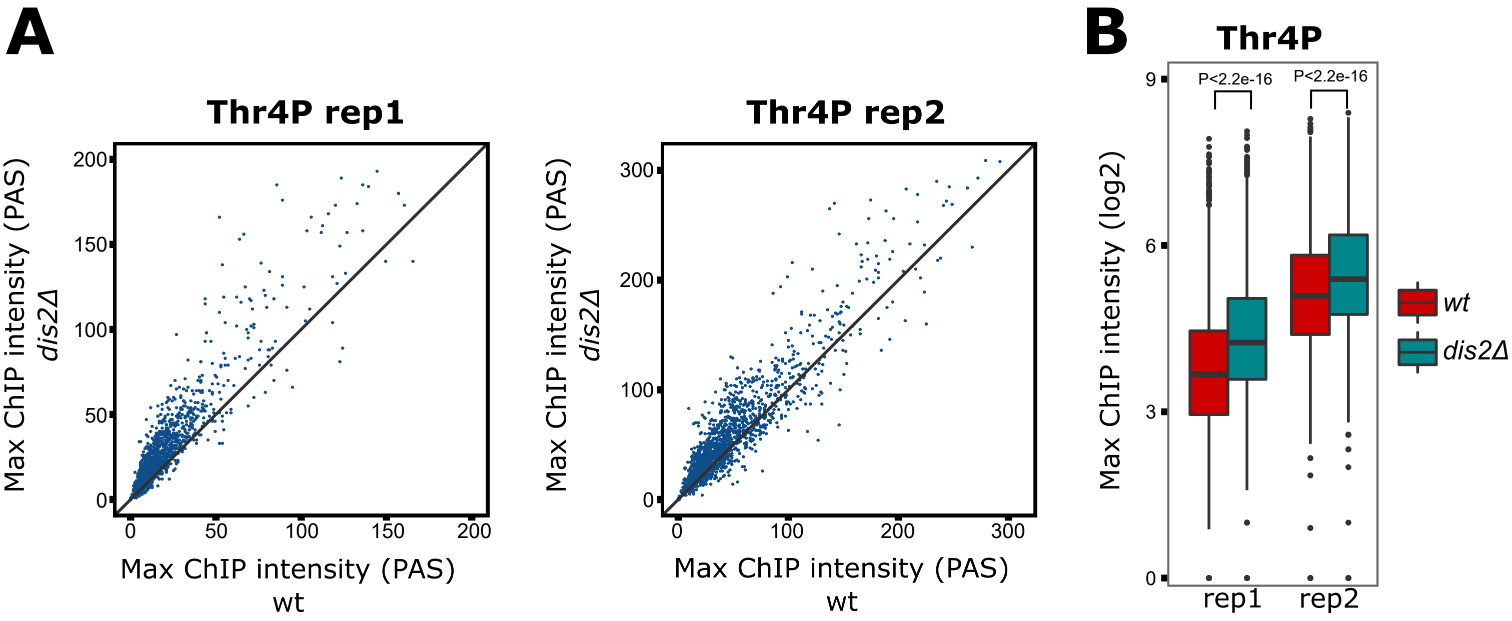

Figure S2. Thr4 ChIP-seq analysis, Related to Figure 3
(A) Scatterplot of Thr4P ChIP signal intensities in wt versus *dis2Δ* over a set of 1735 representative non-overlapping protein-coding genes (see Methods). Duplicate data is presented. Each point corresponds to the spike-in normalized maximum ChIP signal intensity for each gene downstream the PAS. **(B)** Quantitation (boxplots) of Thr4P signal intensities from (A). Duplicate data is presented. p-values (two-sided Wilcoxon rank-sum test) were calculated to test the significance of the difference between wt and *dis2*Δ.

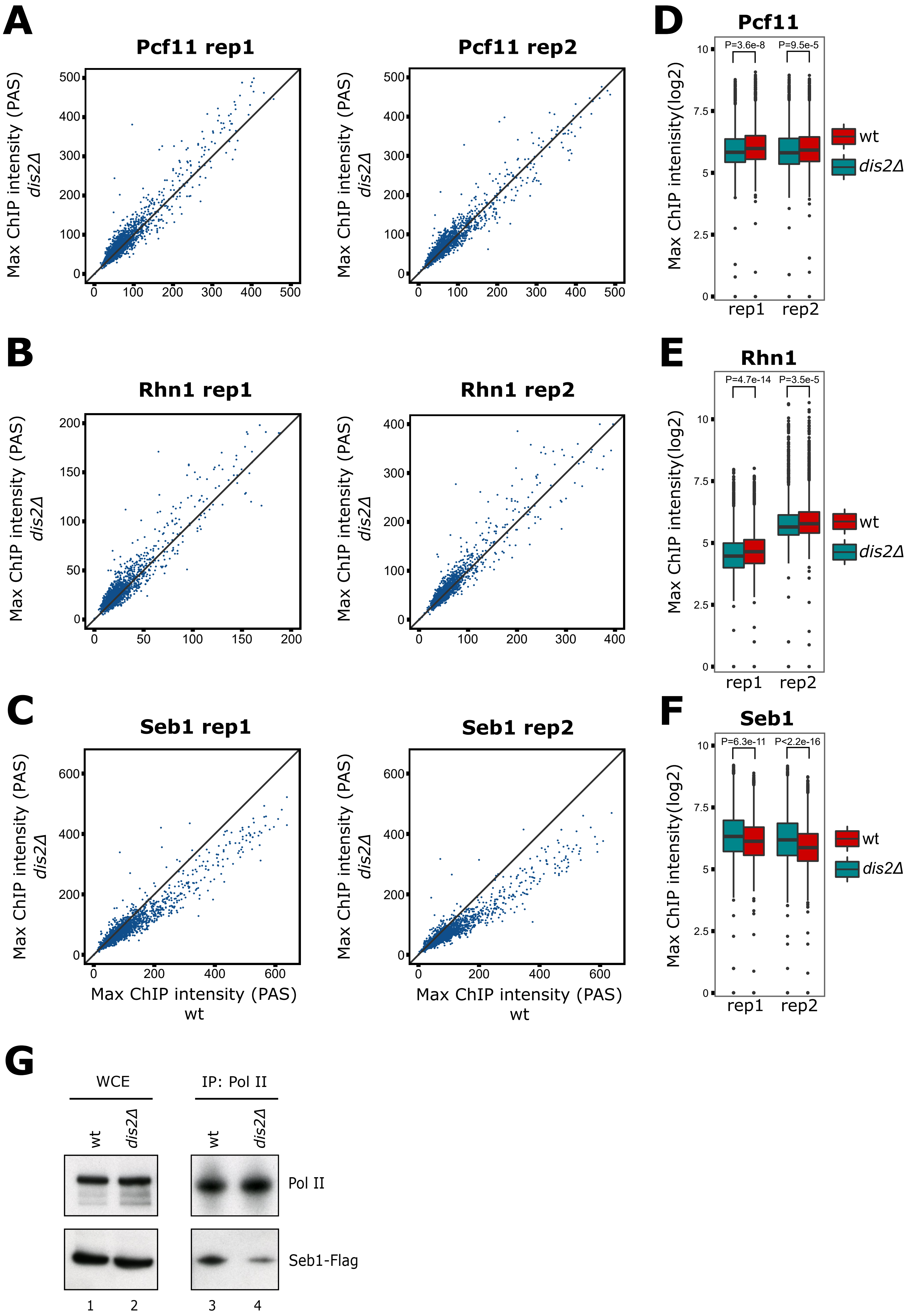
A
Pcf11 rep1
Pcf11 rep2
Max ChIP intensity (PAS) dis2Δ
D
Pcf11
P=3.6e-8
P=9.5e-5
Max ChIP intenisity(log2)
wt
dis2Δ
rep1
rep2
B
Rhn1 rep1
Rhn1 rep2
E
Rhn1
P=4.7e-14
P=3.5e-5
C
Seb1 rep1
Seb1 rep2
Max ChIP intensity (PAS) wt
F
Seb1
P=6.3e-11
P<2.2e-16
G
WCE
IP: Pol II
wt
dis2Δ
Pol II
Seb1-Flag
1
2
3
4

Figure S3. Pcf11, Rhn1 and Seb1 ChIP-seq analysis, Related to Figure 4
(A-C) Scatterplot of Pcf11, Rhn1 and Seb1 ChIP signal intensities in wt and *dis2*Δ. Duplicate data is presented. Protein-coding genes for analysis were selected as above (n=1735). The maximum ChIP signal intensity was calculated for each gene downstream the PAS. **(D-F)** Quantitation (box plots) of Pcf11, Rhn1 and Seb1 ChIP signal intensities from (A-C). Duplicate data is presented. p-values (two-sided Wilcoxon rank-sum test) were calculated to test the significance of the difference between wt and *dis2*Δ. **(G)** Binding of Seb1-Flag to Pol II in wt and *dis2*Δ was determined by immunoprecipitation of Pol II (8WG16), followed by Western blotting with anti-Pol II and Flag antibodies.

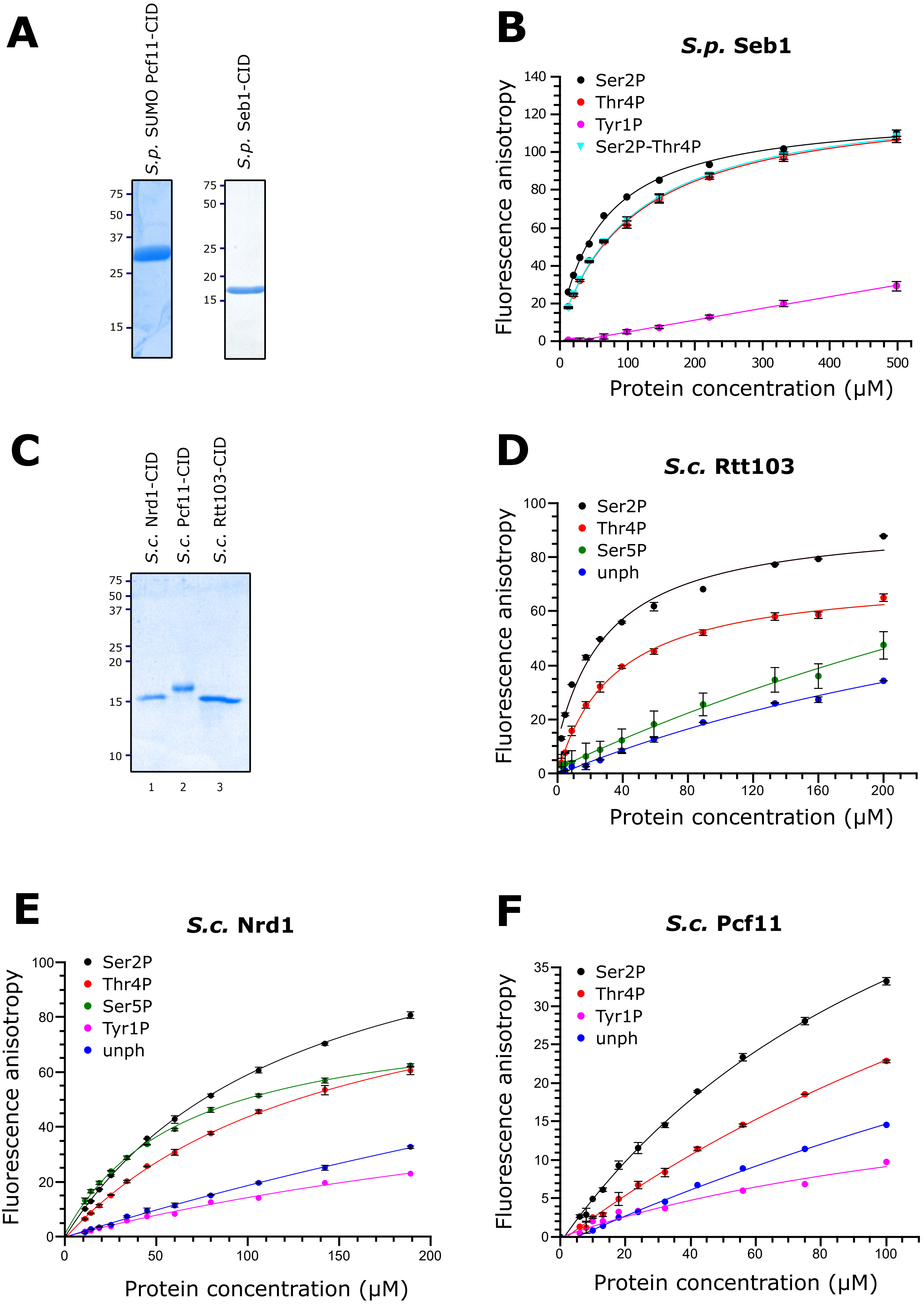

A
S.p. SUMO Pcf11-CID
S.p. Seb1-CID
75
50
37
25
15
75
50
25
20
15
B
S.p. Seb1
Ser2P
Thr4P
Tyr1P
Ser2P-Thr4P
Fluorescence anisotropy
Protein concentration (µM)
C
S.c. Nrd1-CID
S.c. Pcf11-CID
S.c. Rtt103-CID
75
50
37
25
20
15
10
1
2
3
D
S.c. Rtt103
Ser2P
Thr4P
Ser5P
unph
Fluorescence anisotropy
Protein concentration (µM)
E
S.c. Nrd1
Ser2P
Thr4P
Ser5P
Tyr1P
unph
Fluorescence anisotropy
Protein concentration (µM)
F
S.c. Pcf11
Ser2P
Thr4P
Tyr1P
unph
Fluorescence anisotropy
Protein concentration (µM)

Figure S4. Fluorescence anisotropy (FA) assay for yeast termination factors. Related to Figure 4
(A) SDS-PAGE showing the preparations for *S.pombe* SUMO Pcf11-CID and Seb1-CID. **(B)** Binding of *S. pombe* Seb1 to a FAM-tagged two-repeat Ser2P-Thr4P phosphorylated CTD peptide measured by FA (for Ser2P, K_d=67.8 ± 6.8 µM; Thr4P, K_d=114.2 ± 8.4 µM; Ser2P+Thr4P, K_d=112.3 ± 6.6 µM). **(C)** SDS-PAGE showing the preparations for *S. cerevisiae* Rtt103-CID, Nrd1-CID and Pcf11-CID that were utilised for FA assays. **(D-F)** FA assay for *S. cerevisiae* CID containing factors from (C). Error bars show the standard deviation of two technical repeats.

A

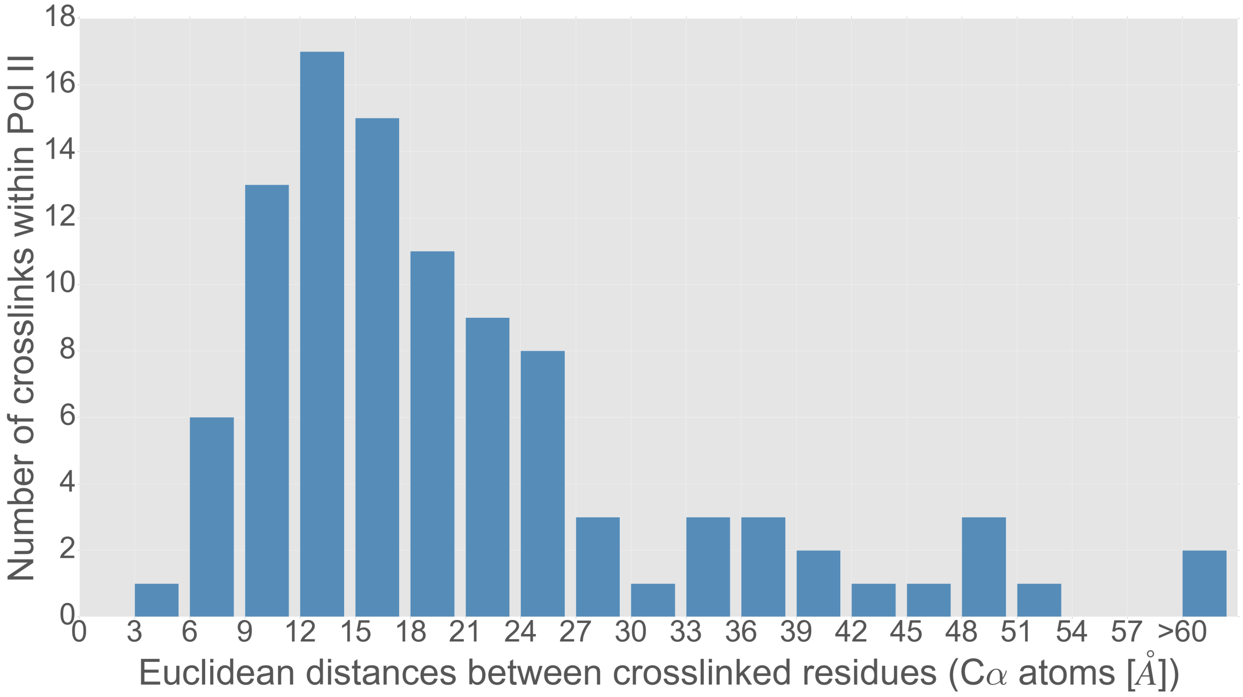

B

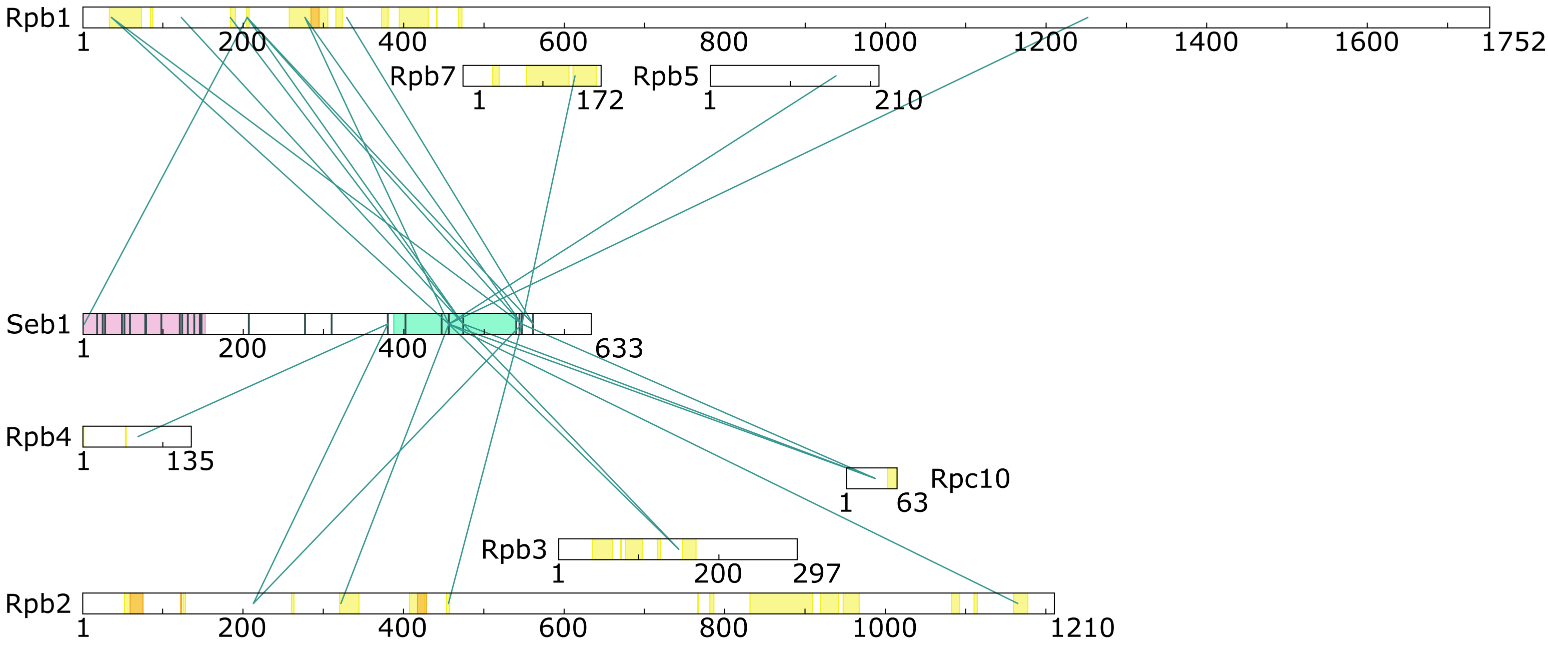

Figure S5. Cross-linking data validation based on Pol II structure, Related to Figure 5
(A) Histogram of distances between Cα atoms of cross-linked residues within Pol II structure (PDB ID: 3H0G). **(B)** Schematic map of the identified lysine-lysine cross-links of the Pol II-Seb1 complex. Lysines of Seb1 are represented as vertical lines. CID and RRM domains of Seb1 are highlighted in pink and green colour, respectively. Regions of predicted contact between fission yeast Pol II and Spt4 and 5 are depicted as orange and yellow boxes.

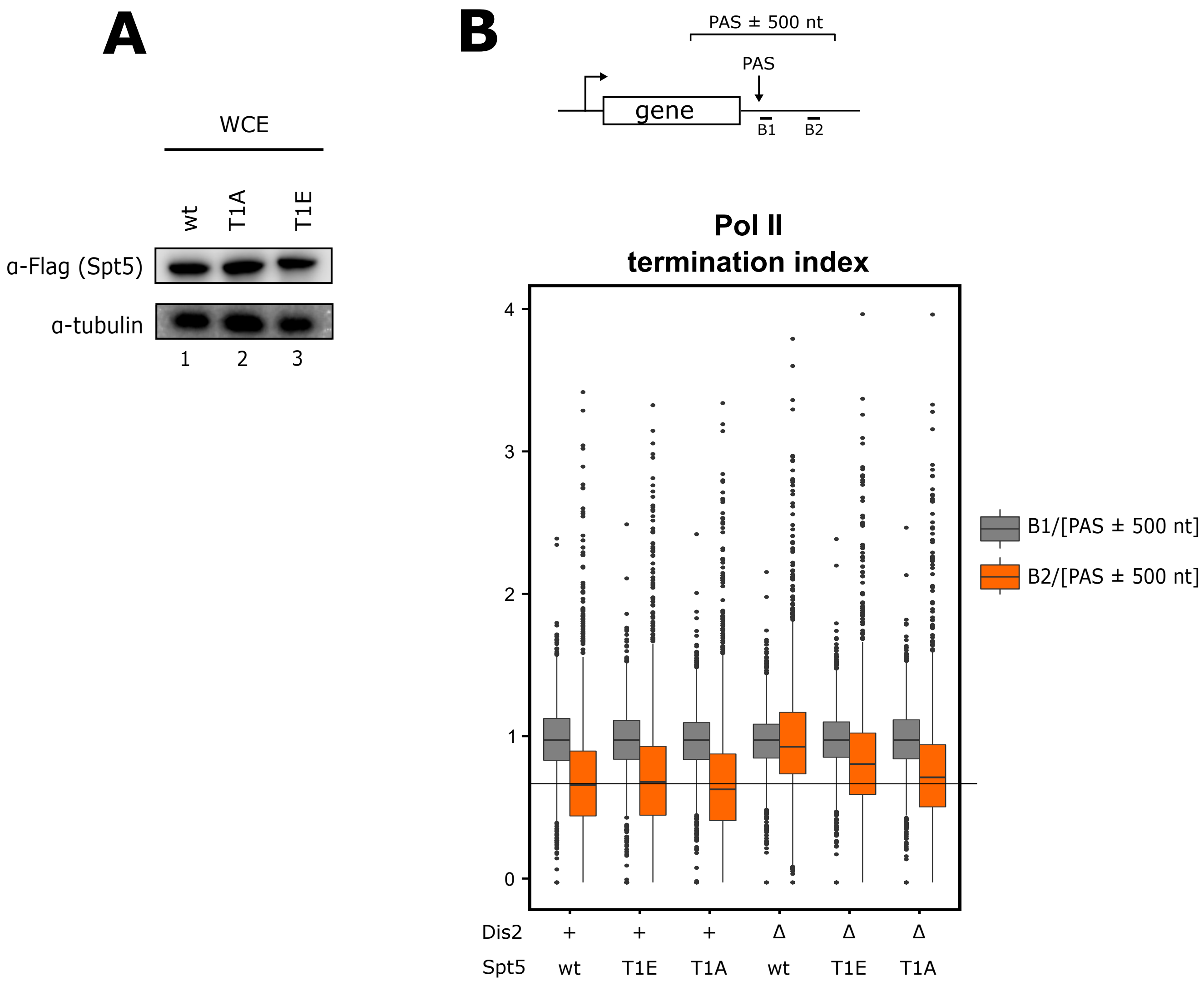

Figure S6. Read-through analysis in *spt5* mutants, Related to Figure 6
(A) Western blot analysis of *spt5* wt/T1A/T1E in whole cell extract. **(B)** Pol II termination index was determined by dividing mapped reads in window B1 or B2 by the read count within [PAS ±500 nt]. Protein-coding genes for analysis were selected as above (n=1735). B1 corresponds to the region from PAS to PAS+50 nt. B2 corresponds to the region from PAS+350 nt to PAS+400 nt.

Table S1: K_d values of CID–CTD interactions, Related to Figure 4 and S4.

	K_d (μM)				
	***S.p.* Pcf11**	***S.p.* Seb1**	***S.c.* Nrd1**	***S.c* .Pcf11**	***S.c.* Rtt103**
Double-repeated Ser2P	48.1 ± 2.7	72.3 ± 6.4	126 ± 4	127 ± 15	32.3 ± 6.8
Double-repeated Thr4P	51.1 ± 5.2	153.2 ± 12.1	148 ± 7	269 ± 57	34.8 ± 3.2
Double-repeated Ser5P	-	280.9 ± 23.1	65.8 ± 3	-	>1000

Table S3: Yeast strains used in this study, Related to STAR Methods.

Strain name	Organism	Genotype	Reference
YP144	*S.pombe*	h+, leu1-32, ura4-Δ18, ade6-M216; his3Δ::1	Lemieux et al., 2011
YP602	*S.pombe*	h+, leu1-32, ura4-Δ18, ade6-M216; his3Δ::1; Rpb9-3xFLAG::kanMX; Rpb1-TEV-CTD (a TEV cleavage site was inserted upstream the CTD)	This study
YP606	*S.pombe*	h-, leu1; ura4; dis2::ura4+	National BioResource Project (NBRP), Japan
YP611	*S.pombe*	h-; leu1; dis2-11	National BioResource Project (NBRP), Japan
YP637	*S.pombe*	h+; leu1-32; ura4-Δ18; ade6-M216; his3Δ::1; Rpb9-3xFLAG::kanMX	This study
YP732	*S.pombe*	h+; leu1-32; ura4-Δ18; ade6-M216; his3Δ::1; dis2::ura4+	This study
YP781	*S.pombe*	h+; leu1-32; ura4-Δ18; ade6-M216; his3Δ::1; Seb1-3XHA-TAP::kanMX	This study
YP782	*S.pombe*	h+; leu1-32; ura4-Δ18; ade6-M216; his3Δ::1; dis2::ura4+; Seb1-3XHA-TAP::kanMX	This study
YP783	*S.pombe*	h+; leu1-32; ura4-Δ18; ade6-M216; his3Δ::1; Pcf11-TAP::kanMX	This study
YP784	*S.pombe*	h+; leu1-32; ura4-Δ18; ade6-M216; his3Δ::1; dis2::ura4+; Pcf11-TAP::kanMX	This study
YP822	*S.pombe*	h+; leu1-32; ura4-Δ18; ade6-M216; his3Δ::1; Rhn1-3XHA-TAP::kanMX	This study
YP823	*S.pombe*	h+: leu1-32; ura4-Δ18; ade6-M216; his3Δ::1; dis2::ura4+; Rhn1-3XHA-TAP::kanMX	This study
YP839	*S.pombe*	h+; leu1-32; ura4-Δ18; ade6-M216; his3Δ::1; Seb1-3XFlag::kanMX	This study
YP840	*S.pombe*	h+: leu1-32; ura4-Δ18; ade6-M216; his3Δ::1; dis2::ura4+; Seb1-3XFlag::kanMX	This study
YP910	*S.pombe*	h+; leu1-32; ura4-Δ18; ade6-M216; his3Δ::1; Seb1-3XHA-TAP::kanMX; Spt5-wt-3xFlag::natMX	This study
YP911	*S.pombe*	h+; leu1-32; ura4-Δ18; ade6-M216; his3Δ::1; Seb1-3XHA-TAP::kanMX; Spt5-T1E-3xFlag::natMX	This study
YP912	*S.pombe*	h+; leu1-32; ura4-Δ18; ade6-M216; his3Δ::1; Seb1-3XHA-TAP::kanMX; Spt5-T1A-3xFlag::natMX	This study
YP913	*S.pombe*	h+; leu1-32; ura4-Δ18; ade6-M216; his3Δ::1; dis2::ura4+; Seb1-3XHA-TAP::kanMX; Spt5-wt-3xFlag::natMX	This study
YP914	*S.pombe*	h+; leu1-32; ura4-Δ18; ade6-M216; his3Δ::1; dis2::ura4+; Seb1-3XHA-TAP::kanMX; Spt5-T1E-3xFlag::natMX	This study
YP915	*S.pombe*	h+; leu1-32; ura4-Δ18; ade6-M216; his3Δ::1; dis2::ura4+; Seb1-3XHA-TAP::kanMX; Spt5-T1A-3xFlag::natMX	This study
YP956	*S.pombe*	h+; leu1-32; ura4-Δ18; ade6-M216; his3Δ::1; Spt5-wt-3xFlag::natMX	This study
YP957	*S.pombe*	h+; leu1-32; ura4-Δ18; ade6-M216; his3Δ::1; Spt5-T1A-3xFlag::natMX	This study
YP958	*S.pombe*	h+; leu1-32; ura4-Δ18; ade6-M216; his3Δ::1; Spt5-T1E-3xFlag::natMX	This study
YP959	*S.pombe*	h+; leu1-32; ura4-Δ18; ade6-M216; his3Δ::1; dis2::ura4+; Spt5-wt-3xFlag::natMX	This study
YP960	*S.pombe*	h+; leu1-32; ura4-Δ18; ade6-M216; his3Δ::1; dis2::ura4+; Spt5-T1A-3xFlag::natMX	This study
YP961	*S.pombe*	h+; leu1-32; ura4-Δ18; ade6-M216; his3Δ::1; dis2::ura4+; Spt5-T1E-3xFlag::natMX	This study
YP1029	*S.pombe*	h-/h+; ade6-M210/ade6-M216; arg3-Δ4/arg3-Δ4; ura4-Δ18/ura4-Δ18; his3-Δ1/his3-Δ1; rpb1-wt/rpb1-T4A-KanMX6	This study

YP1083	*S.pombe*	h-/h+; ade6-M210/ade6-M216; arg3-Δ4/arg3-Δ4; ura4-Δ18/ura4-Δ18; his3-Δ1/his3-Δ1; rpb1-wt/rpb1-T4E-KanMX6	This study
YF336	*S.cerevisiae*	MATa; his3Δ1; leu2Δ0; met15Δ0; ura3Δ0	Kim et al., 2004
YF1429	*S.cerevisiae*	MATa; his3Δ1; leu2Δ0; met15Δ0; ura3Δ0; Pcf11-TAP::His3	Ghaemmaghami et al., 2003
